# Supplementary material for: Cooperative spin crossover leading to bistable and multi-inert system states in an iron(III) complex
Source: Nat Commun. 2024 Aug 25;15:7321. doi: 10.1038/s41467-024-51675-1 (PMC11345420; doi:10.1038/s41467-024-51675-1)
Supplement: Supplementary file 1 — Supplementary Information [file 41467_2024_51675_MOESM1_ESM.pdf]

Cooperative spin crossover leading to bistable and multi-inert system states in an iron(III) complex

SUPPLEMENTARY INFORMATION

Authors

Andreas Dürrmann,[1,2] Gerald Hörner,[1,2] Dirk Baabe,[3] Frank W. Heinemann,[4] Mauricio A. C. de Melo,[5] and Birgit Weber*[1,2]

Affiliations

[1] A. Dürrmann, Dr. G. Hörner, Prof. Dr. B. Weber
Main body of experimental work was performed at: Inorganic Chemistry IV, University of Bayreuth, Universitätsstraße 30, 95447 Bayreuth (Germany)

[2] A. Dürrmann, Dr. G. Hörner, Prof. Dr. B. Weber
New permanent address: Institute for Inorganic and Analytical Chemistry, Friedrich Schiller University Jena, Humboldtstraße 8, 07743 Jena (Germany), E-mail: andreas.duerrmann@uni-jena.de, gerald.hoerner@uni-jena.de, birgit.weber@uni-jena.de

[3] Dr. D. Baabe
Institut für Anorganische und Analytische Chemie, Technische Universität Braunschweig, Hagenring 30, 38106 Braunschweig (Germany), E-mail: d.baabe@tu-braunschweig.de

[4] Dr. F. W. Heinemann
Lehrstuhl für Anorganische und Allgemeine Chemie, Friedrich-Alexander-University Erlangen-Nürnberg, Egerlandstr. 1, 91058 Erlangen (Germany), E-mail: frank.heinemann@fau.de

[d] Prof. Dr. M. A. C. de Melo
Departamento de Fisica, Universidade Estadual de Maringá, 87020-900 Maringa - PR (Brazil), E-mail: mmelo@dfi.uem.br

TABLE OF CONTENTS

1 Supplementary Note 1: Theoretical background 1
2 Synthetic background 3
3 Single crystal X-ray diffraction 4
3.1 Crystallographic details of ligand HL 4
3.2 Crystallographic details of compound FeB 6
3.3 Hirshfeld surface analysis 11
3.4 Crystallographic Figures 12
3.5 Powder diffraction patterns of compound FeB 17
4 SQUID magnetometry 19
4.1 Scan rate dependent spin crossover 19
4.1.1 Run 1 21
4.1.2 Run 2 23
4.1.3 Run 3 26
4.1.4 Run 4 28
4.1.5 Run 5 30
4.2 Relaxation dynamics 33
5 ^{57}Fe Mössbauer spectroscopy 35
6 Further characterisation 43
6.1 Mass spectra of compound FeB 43
6.2 IR spectrum of compound FeB 44
6.3 ^{1}H NMR spectrum of ligand HL 45
7 Supplementary references 46

1 SUPPLEMENTARY NOTE 1: THEORETICAL BACKGROUND

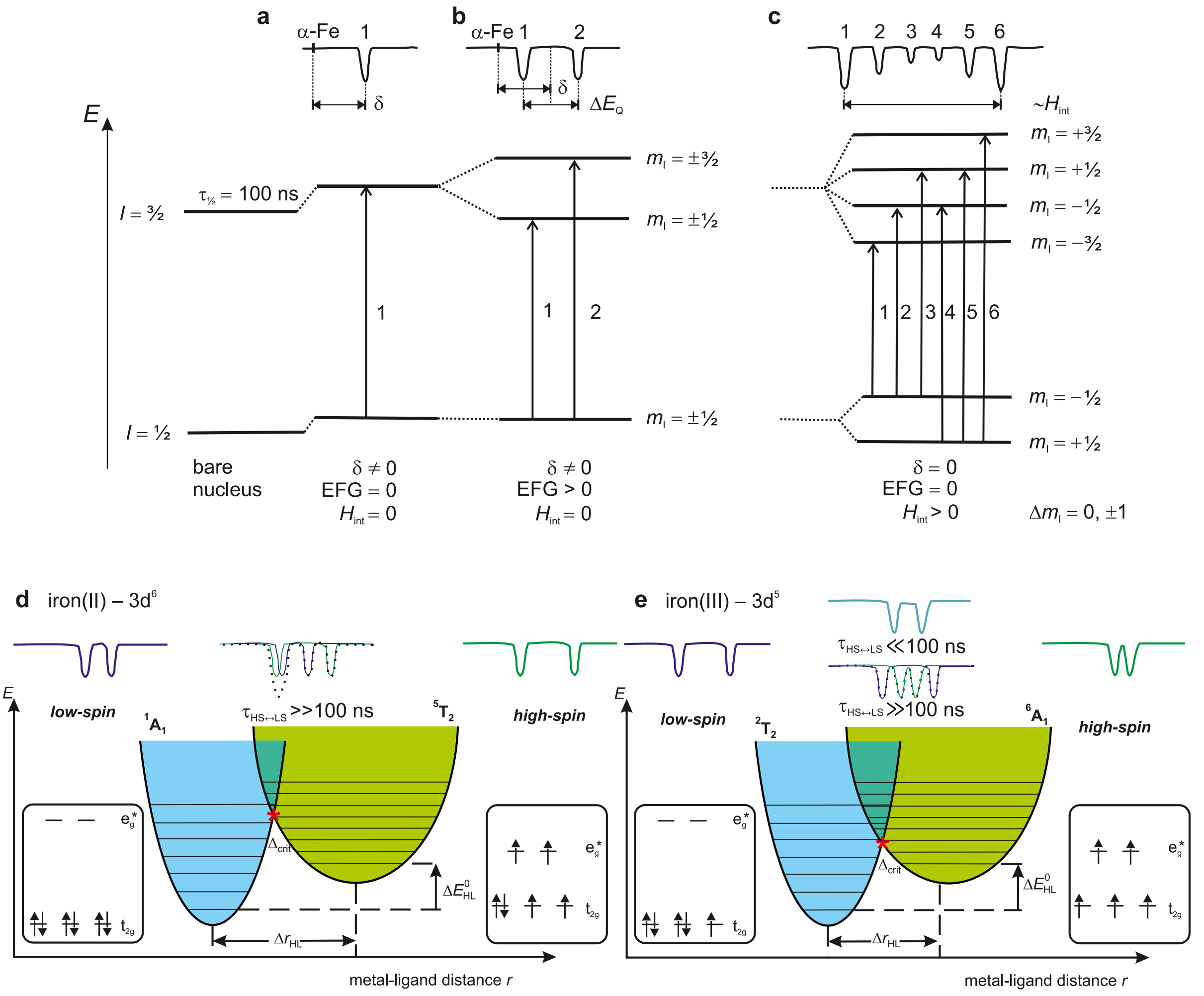

Supplementary Figure 1 | Very basic concepts of ^{57}Fe Mössbauer spectroscopy and thermal spin crossover (SCO). a Electric monopole interaction between the nucleus and electron density (origin of isomeric shift δ).[1,2] **b** Electric quadrupole interaction between the nucleus in the excited state (its electric quadrupole moment) and an (inhomogeneous) electric field located at the nucleus (described by the electric field gradient).[3,4] **c** Magnetic dipole splitting: Interaction between the nuclear dipole moment and a magnetic field H.[5] **d,e** Potential well diagram and ligand field splitting for iron(II/III) in the context of thermally induced spin crossover (SCO).[6] The potential wells are drawn in the harmonic approximation for the sake of simplicity.

In Mössbauer spectroscopy, small changes in the energy separation between the nuclear ground state (I = ½) and the nuclear excited state (I = 3/2) of the ^{57}Fe nuclei of a sample are detected. A given Mössbauer spectrum can formally be described with a set of parameters. Beside the width of the absorption lines, for many chemical applications, this set is composed of three quantities, *i.e.*, the isomer shift δ, the quadrupole splitting ΔE_Q and the internal magnetic (hyperfine) field H_{int}. The isomer shift δ (Supplementary Figure 1a), which is typically

compared to the isomer shift of a reference material (here α-Fe), is caused by (small) differences in the electronic environment around the nucleus, *e.g.*, due to differences in the spin and/or oxidation state. The $I = 3/2$ excited state possesses a nuclear electric quadrupole moment. In combination with an electric field gradient (EFG), which is induced by the surrounding electronic environment (*e.g.*, due to an electron distribution that differs from spherical symmetry and/or a coordination environment that differs from cubic symmetry), an energetic splitting of the $I = 3/2$ m_I-states (corresponding to $m_I = \pm 1/2$ and $m_I = \pm 3/2$) is obtained, leading to the experimental observation of a doublet with the quadrupole splitting ΔE_Q (Supplementary Figure 1b). In the case of (octahedral) iron(II) low-spin (LS) and iron(III) high-spin (HS) complexes, the very symmetric electron distribution among the d-orbitals leads to a very small to negligible quadrupole splitting, while for HS iron(II) and LS iron(III) the electron distribution is less symmetric and ΔE_Q is thus larger, as schematically shown in Supplementary Figure 1d and e. In the presence of an internal magnetic (hyperfine) field, the m_I-degeneracy of the $I = 1/2$ ground state and the $I = 3/2$ excited state of a ^{57}Fe nucleus will be completely removed, leading to six allowed transitions (Supplementary Figure 1c).

The term spin crossover describes the transition between two different electronic states, most often observed for $3d^{4-7}$ metal complexes in an octahedral coordination environment, that can be triggered by different physical stimuli, *i.e.*, by change in temperature, pressure, or light irradiation or by chemical stimuli, *e.g.*, host-guest interactions. Precondition is an energetically favoured low-spin state (blue potential well in Supplementary Figure 1d and e), while the high-spin state is entropically favoured due to a higher degree of vibrational disorder (green potential well in Supplementary Figure 1d and e). Accordingly, the HS state dominates at higher temperatures, while the LS state is preferred at lower temperatures. The associated changes in the occupation of the antibonding e_g* orbitals lead to different Fe–L bond lengths in the HS and the LS state. Those changes are more pronounced in the case of iron(II) complexes compared to iron(III) complexes. As a consequence, the crossing point Δ_{crit} of the HS and LS potential wells, relevant for the rate constant of the HS↔LS interconversion at a given temperature in the crossover region, is energetically less favourable in the case of iron(II) compared to iron(III) systems. The slow HS↔LS interconversion for iron(II) systems leads to the observation of two separate doublets for the HS and the LS state in the crossover region (Supplementary Figure 1d). In the case of iron(III), the faster spin interconversion

between the two states can result in convoluted Mössbauer spectra in the crossover region, strongly depending on the system investigated. A very fast HS↔LS relaxation compared to the lifetime of the excited $I = 3/2$ state ($\tau_{HS \leftrightarrow LS} \ll 100$ ns) leads to one time-averaged signal with averaged δ and ΔE_Q (Supplementary Figure 1e), while slow HS↔LS relaxation ($\tau_{HS \leftrightarrow LS} \gg 100$ ns) can again lead to two separate signals like in the case of iron(II).

2 SYNTHETIC BACKGROUND

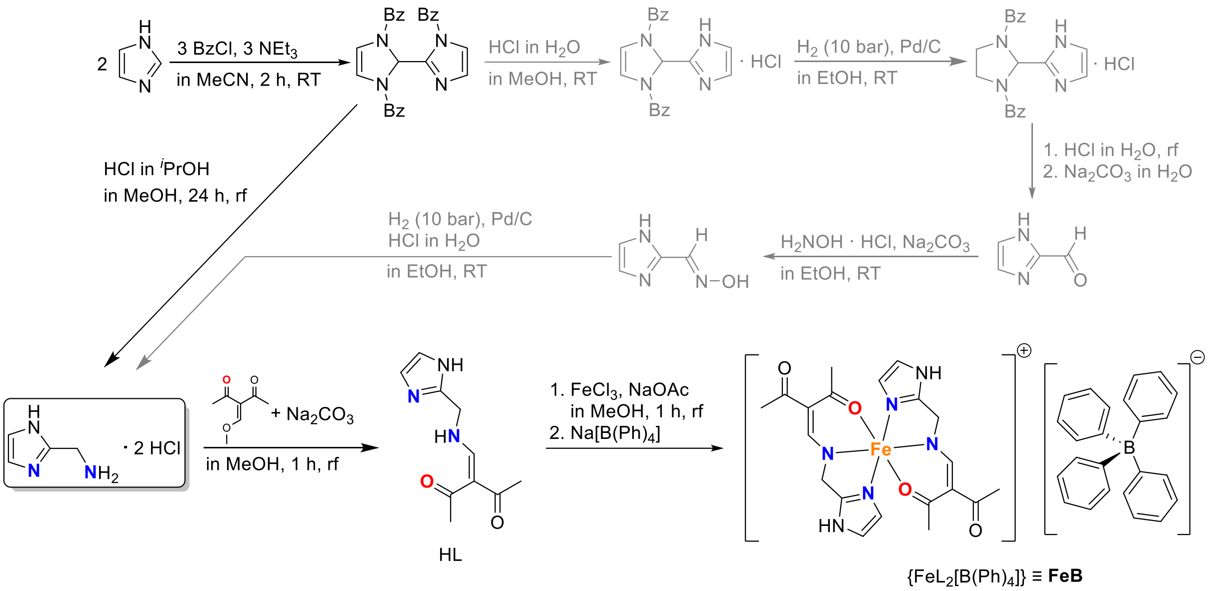

Supplementary Figure 2 | Synthetic approach towards compound FeB. The key intermediate (1*H*-imidazol-2-yl)methanamine dihydrochloride is highlighted (bottom left).[7] Bz denotes benzoyl groups. Molecular sketches in grey show an alternative route which did not give reliable results in our attempts.[8]

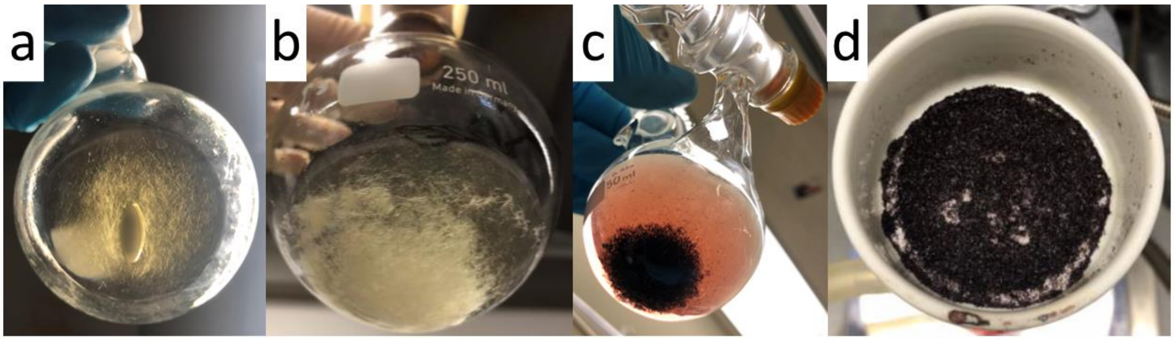

Supplementary Figure 3 | a, b Snapshot of the crystallising ligand. **c, d** Snapshot of the crystallising/isolated compound **FeB.**

3 SINGLE CRYSTAL X-RAY DIFFRACTION

3.1 Crystallographic details of ligand HL

Crystals of HL that were suitable for X-ray structure analysis could be obtained upon cooling a concentrated acetone solution.

Supplementary Table 1 | Crystallographic data of the ligand HL.

	HL
CCDC number	2210063
Formula	$C_{10}H_{13}N_3O_2$
Sum formula	$C_{10}H_{13}N_3O_2$
M / g mol^{-1}	207.23
Crystal system	orthorhombic
Space group	*Pccn* (No. 56)
Crystal description	colourless needle
Crystal size / mm	0.10 × 0.11 × 0.31
a / Å	14.8966(6)
b / Å	18.7832(7)
c / Å	7.1250(2)
α / °	90
β / °	90
γ / °	90
V / Å^3	1993.62(12)
Z	8
$\rho_{calculated}$ / g cm^{-3}	1.381
μ / mm^{-1}	0.099
F(000)	880
θ_{range} / °	1.7–28.5
Index range (h k l)	$-19 \leq h \leq 19$<br>$-24 \leq k \leq 24$<br>$-4 \leq l \leq 9$
Radiation	Mo-K_α (λ = 0.71073 Å)
T / K	200
Measured reflections	23739
Independent reflections	2462
Reflections with $I > 2\sigma(I)$	1509
R_{int}	0.09
Restraints and parameters	0 and 141
R_1 [$F^2 > 2\sigma(F^2)$]	0.0569
wR_2(F^2)	0.1597
GooF (S)	1.02
$\rho_{residual}$ (largest peak and hole) / eÅ^{-3}	0.28, –0.20

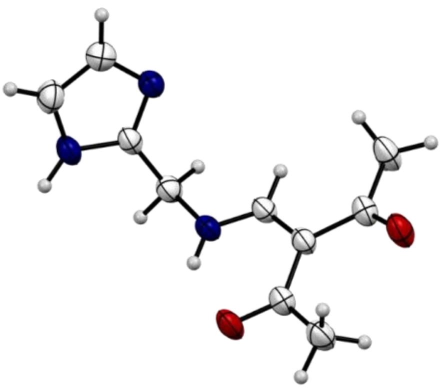

Supplementary Figure 4 | Molecular structure of ligand HL (asymmetric unit). Displacement ellipsoids are drawn at 50 % probability level.

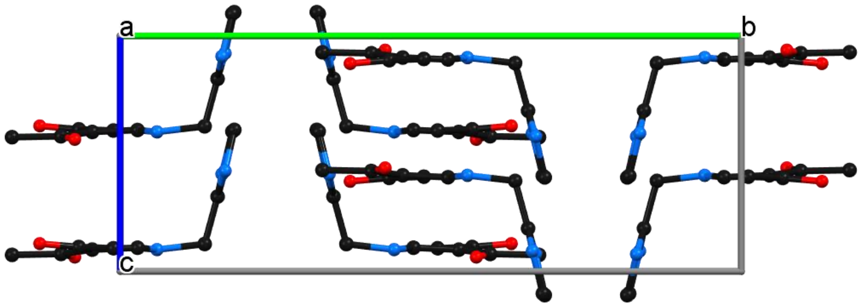

Supplementary Figure 5 | Excerpt of the crystal packing of HL along the crystallographic axis *a*. Hydrogen atoms were omitted for clarity.

3.2 Crystallographic details of compound FeB

Supplementary Table 2 | Crystallographic data of compound **FeB**.

	**FeB**-HS	**FeB**-LS
CCDC number	2210430	2210431
Formula	$C_{20}H_{24}FeN_6O_4$, $C_{24}H_{20}B$	$C_{20}H_{24}FeN_6O_4$, $C_{24}H_{20}B$
Sum formula	$C_{44}H_{44}BFeN_6O_4$	$C_{44}H_{44}BFeN_6O_4$
M / g mol^{-1}	787.51	787.51
Crystal system	orthorhombic	orthorhombic
Space group	*Pbcn* (No. 60)	*Pbcn* (No. 60)
Crystal description	red column	red column [a]
Crystal size / mm	0.36 × 0.08 × 0.08	0.36 × 0.08 × 0.08
a / Å	16.3327(6)	17.0583(7)
b / Å	15.7996(6)	15.3458(6)
c / Å	15.0588(6)	14.6767(6)
α / °	90	90
β / °	90	90
γ / °	90	90
V / Å^3	3885.9(3)	3842.0(3)
Z	4	4
$\rho_{calculated}$ / g cm^{-3}	1.346	1.361
μ / mm^{-1}	0.440	0.445
F(000)	1652	1652
θ_{range} / °	1.7–27.9	1.7–26.1
Index range (h k l)	$-21 \le h \le 18$<br>$-20 \le k \le 20$<br>$-19 \le l \le 16$	$-21 \le h \le 21$<br>$-18 \le k \le 18$<br>$-17 \le l \le 16$
Radiation	Mo-K_α (λ = 0.71073 Å)	Mo-K_α (λ = 0.71073 Å)
T / K	120	65
Measured reflections	70021	54808
Independent reflections	4610	3742
Reflections with $I > 2\sigma(I)$	2496	2011
R_{int}	0.125	0.198
Restraints and parameters	0 and 259	0 and 259
R_1 [$F^2 > 2\sigma(F^2)$]	0.0508	0.0689
w$R_2(F^2)$	0.1077	0.1314
GooF (S)	0.998	1.119
$\rho_{residual}$ (largest peak and hole) / eÅ^{-3}	0.455, –0.740	0.734, –0.689

[a] The experimental setup (He cryostate) did not allow for observation of any colour change.

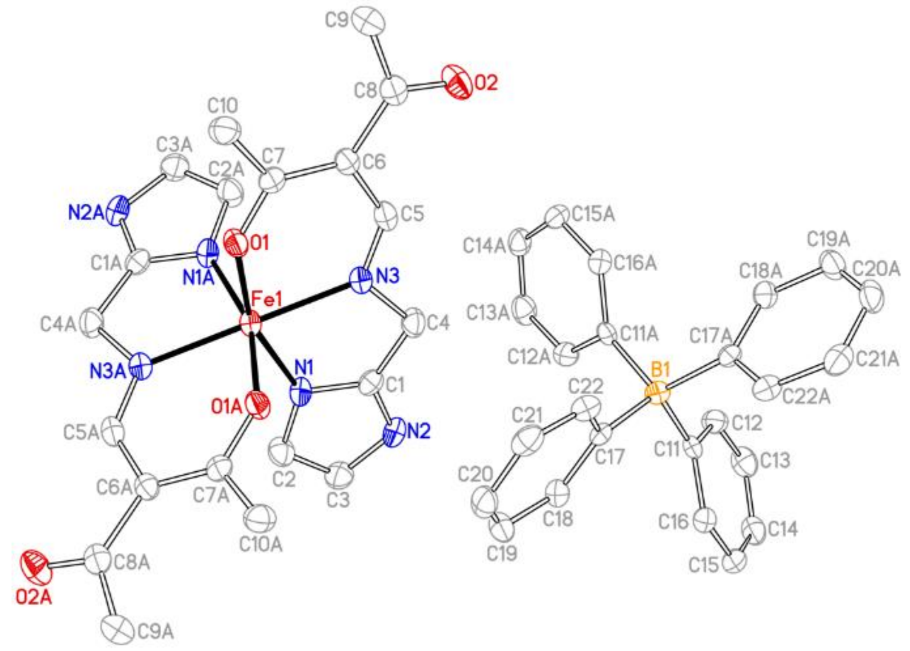

Supplementary Figure 6 | Molecular structure of FeB-HS with complete numbering. Displacement ellipsoids are drawn at 50 % probability level. Hydrogen atoms are omitted for clarity. The letter A represents atoms that are created by symmetry (asymmetric unit).

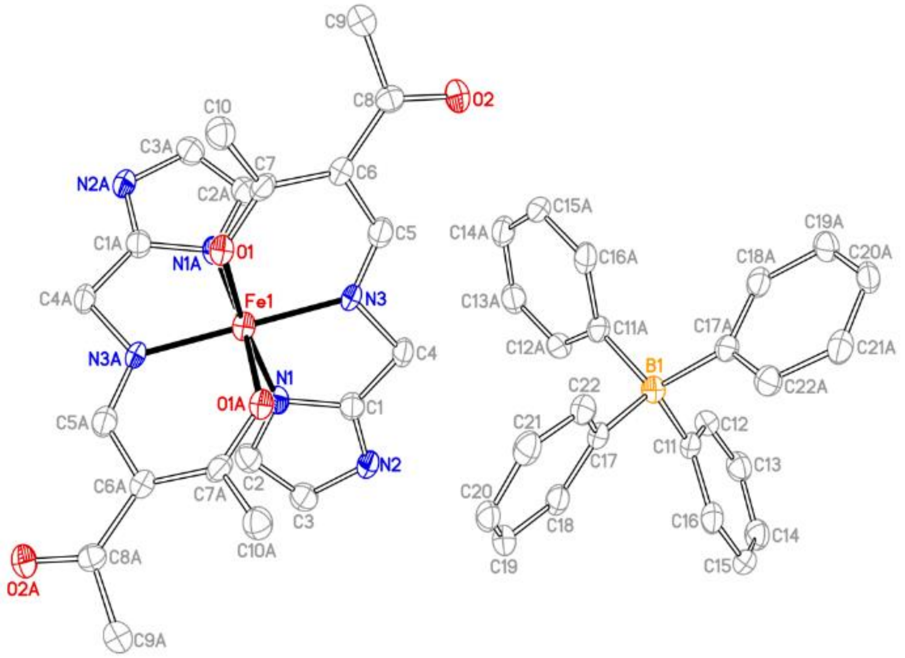

Supplementary Figure 7 | Molecular structure of FeB-LS with complete numbering. Displacement ellipsoids are drawn at 50 % probability level. Hydrogen atoms are omitted for clarity. The letter A represents atoms that are created by symmetry (asymmetric unit).

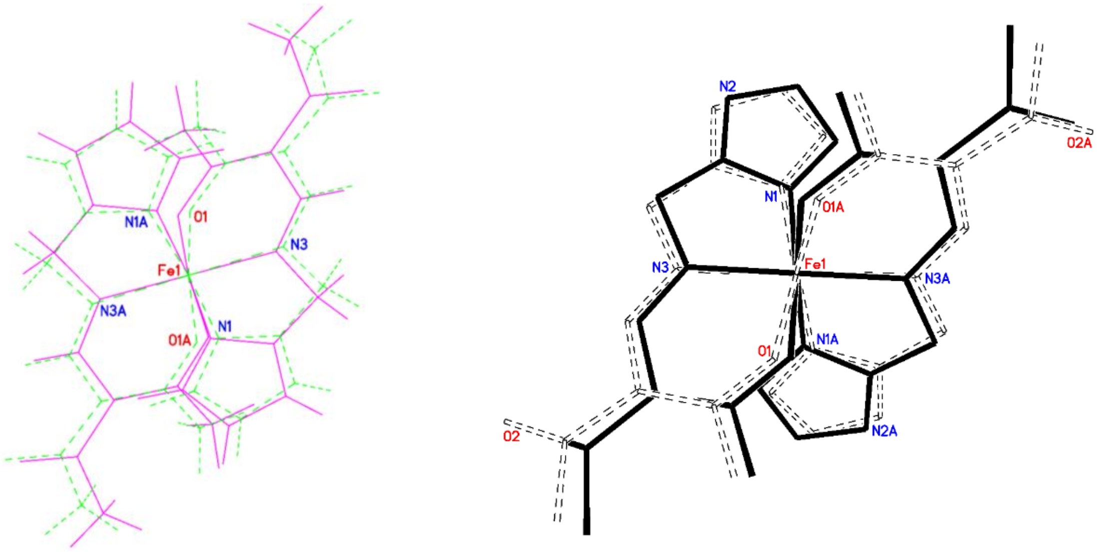

Supplementary Figure 8 | Structural overlays of the complex cation at $T = 65$K (LS, solid line) and $T = 120$K (HS, dashed line) in two different presentations.

Supplementary Table 3 | Atomic distances of the inner coordination sphere of the complex cation and related metrics as well as octahedral distortion parameters of **FeB**-HS and **FeB**-LS.

	d(Fe–N$_{Im}$)	d(Fe–N$_{ax}$)	d(Fe–O)	∡(N$_{Im}$–Fe–O)	V_{cell}	Θ [a]	Σ [b]
$T = 120$ K (HS)	2.075	2.096	1.953	161.09	3885.9	309.35	80.85
$T = 65$ K (LS)	1.937	1.913	1.894	172.16	3842.0	134.30	43.02
|Δ|	0.138 (6.6 %)	0.183 (8.7 %)	0.059 (3.0 %)	11.07 (6.4 %)	43.9 (1.1 %)	175.05	37.83
	Å	Å	Å	°	Å^3	°	°

[a] $\Sigma = \sum_{i=1}^{12} |90 - \alpha_i|$ where α_i corresponds to the 12 *cis* N/O–Fe–N/O angles ∡ (gives information about the octahedral deformation).

[b] $\Theta = \sum_{j=1}^{24} |60 - \theta_j|$ where θ_j corresponds to the 24 torsional angles between donor atoms on opposite triangular faces of the octahedron along its pseudo-threefold axes ("trigonal-twist angle", indicates to which extent the octahedron is distorted with respect to a trigonal prismatic structure).

In case of an ideal octahedral symmetry: $\Sigma = \Theta = 0$.

Calculation is readily accomplished with OctaDist.[9]

Supplementary Table 4 | Metrics of some intermolecular interactions of **FeB**-HS and **FeB**-LS.

	$d(N_{Im}H\cdots O)$	$\measuredangle(N_{Im}H\cdots O)$	$d(CH_2\cdots\pi)$	$\measuredangle(CH_2\cdots\pi)$	$d(C_{Me}H\cdots\pi)$	$\measuredangle(C_{Me}H\cdots\pi)$
T = 120 K (HS)	2.35	120	2.83, 2.80	133, 149	3.03, 3.00	161, 139
T = 65 K (LS)	2.01	139	2.53, 2.63	157, 158	2.88, 2.94	163, 141
|Δ|	0.34<br>(14.5 %)	19<br>(13.7 %)	0.30 (10.6 %)<br>0.17 (6.1 %)	24 (15.3 %)<br>9 (5.7 %)	0.14 (4.6 %)<br>0.06 (2 %)	2 (1 %)<br>2 (1 %)
	Å	°	Å	°	Å	°

Supplementary Table 5 | Summary of selected hydrogen bonds of **FeB** with the corresponding bond lengths *d* and angles ∡.

	d(D–H)	*d*(H···A)	*d*(D···A)	∡(H···A)
		**FeB**-HS		
N2–H2···O2 [a]	0.81(3)	2.35(3)	2.839(3)	120(3)
C3–H3···O2 [a]	0.95	2.54	2.952(3)	106
	Å	Å	Å	°

[a] 0.5 + x, 1.5 – y, 1 – z

	d(D–H)	*d*(H···A)	*d*(D···A)	∡(H···A)
		**FeB**-LS		
N2–H2···O2 [a]	0.88(5)	2.01(5)	2.742(5)	139(4)
C3–H3···O2	0.95	2.859	3.107	96
	Å	Å	Å	°

[a] 0.5 + x, 1.5 – y, 1 – z

Supplementary Table 6 | Summary of selected C–H···π interactions of **FeB**. C_g represents the centroid of the ring number.

C–H	C_g	$d(H\cdots C_g)$	∡(C–H···C_g)	$d(C\cdots C_g)$
		**FeB**-HS		
C4–H4A	C17–C18–C19–C20–C21–C22 [a]	2.80	149	3.684(3)
C4–H4B	C11–C12–C13–C14–C15–C16 [b]	2.83	133	3.584(3)
C9–H9C	C11–C12–C13–C14–C15–C16	3.03	161	3.964
C10–H10C	C17–C18–C19–C20–C21–C22 [c]	3.00	139	3.788(3)
		Å	°	Å

[a] x, y, z
[b] 1 – x, y, 1.5 – z
[c] 1 – x, 1 – y, 1 – z

C–H	C_g	$d(H\cdots C_g)$	∡(C–H···C_g)	$d(C\cdots C_g)$
		**FeB**-LS		
C4–H4A	C11–C12–C13–C14–C15–C16 [a]	2.63	158	3.565(5)
C4–H4B	C17–C18–C19–C20–C21–C22 [b]	2.53	157	3.470(5)
C9–H9C	C11–C12–C13–C14–C15–C16 [c]	2.88	163	3.830(5)
C10–H10C	C17–C18–C19–C20–C21–C22 [d]	2.94	141	3.749(5)
		Å	°	Å

[a] 1 – x, y, 1.5 – z
[b] x, y, z
[c] –0.5 + x, 1.5 – y, 1 – z
[d] 1 – x, 1 – y, 1 – z

3.3 Hirshfeld surface analysis

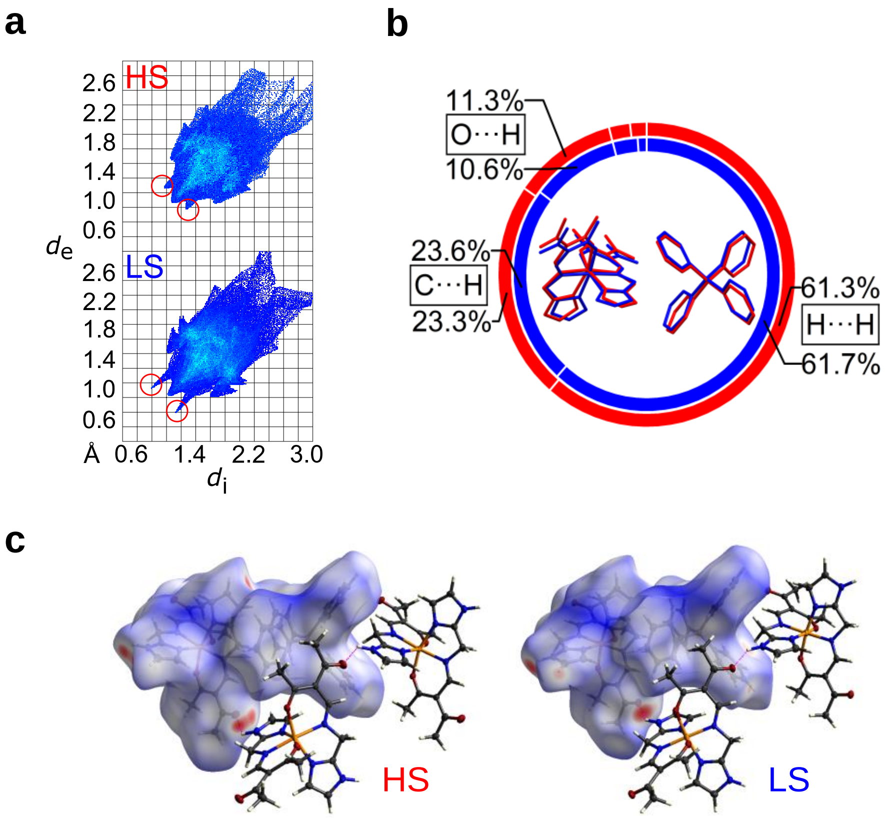

Supplementary Figure 9 | Hirshfeld surface analysis (HSA). HSA is a useful tool to explore the nature of non-covalent interactions.[10,11] **a** 2D fingerprint plots of compound **FeB**. **b** Significant contributions to the Hirshfeld surfaces of **FeB**-HS (red) and **FeB**-LS (blue). The other non-specified contributions are C···N, C···O, and C···C contacts. **c** Corresponding 3D Hirshfeld surfaces mapped over d_{norm} which support the importance of hydrogen bonding (red circles/spots).

3.4 Crystallographic figures

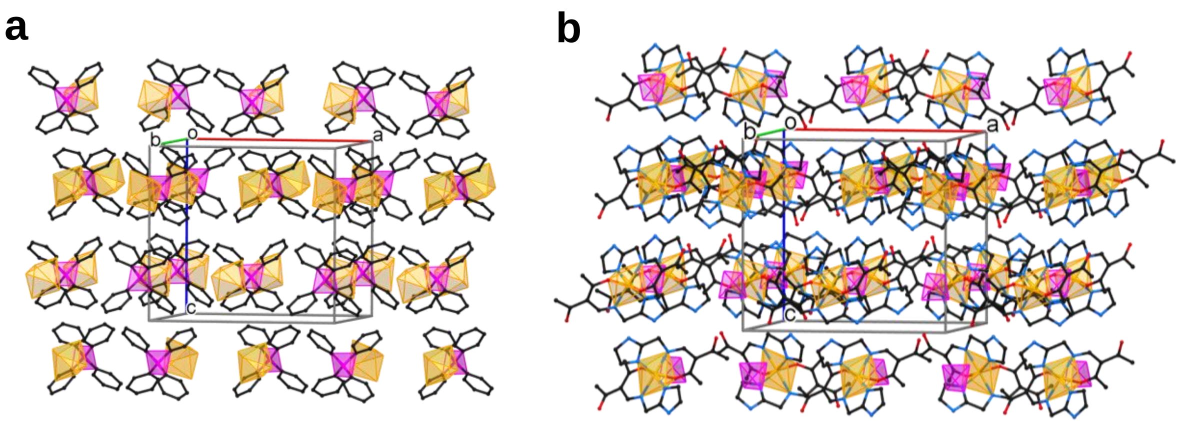

Supplementary Figure 10 | Excerpt of the crystal packing of compound FeB-HS. For the sake of clarity, two different representations are given. **a** Inner coordination sphere (orange pseudo-octahedron) and complete $[B(Ph)_4]^-$ anion. **b** Complete complex cation and simplified anion, shaped as magenta $[BC_4]$ tetrahedron. Hydrogen atoms were omitted in both cases.

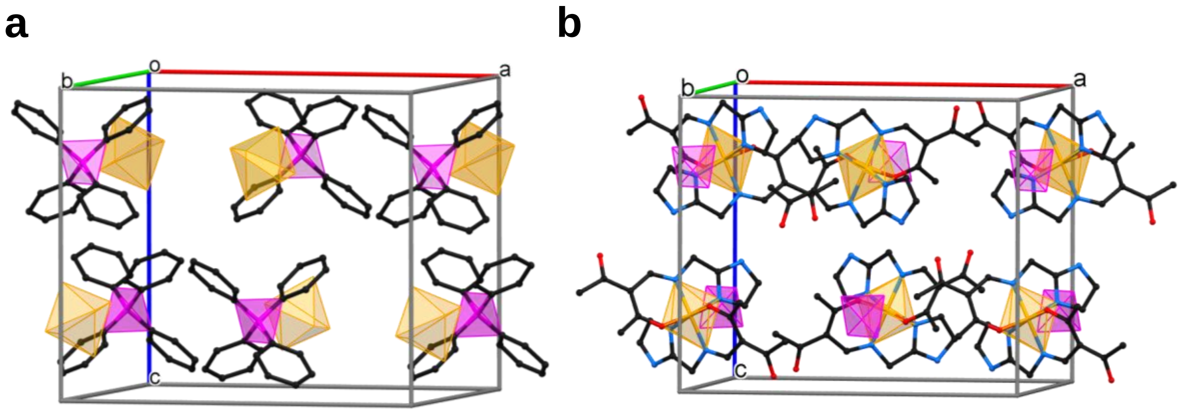

Supplementary Figure 11 | Excerpt of the crystal packing of compound FeB-LS. For the sake of clarity, two different representations are given. **a** Inner coordination sphere (orange pseudo-octahedron) and complete $[B(Ph)_4]^-$ anion. **b** Complete complex cation and simplified anion, shaped as magenta $[BC_4]$ tetrahedron. Hydrogen atoms were omitted in both cases.

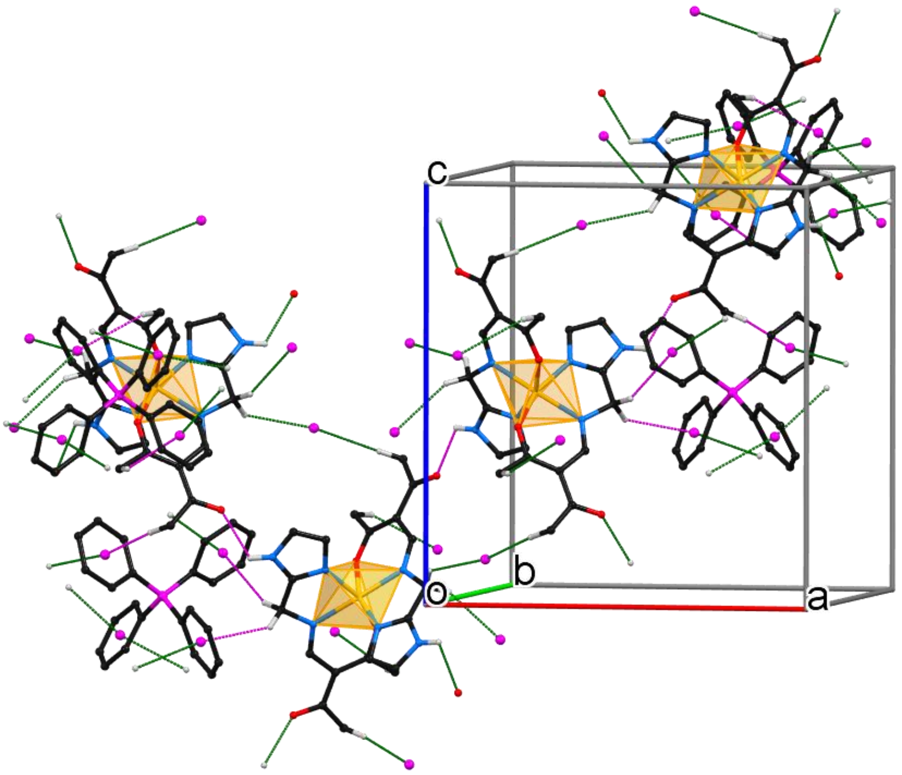

Supplementary Figure 12 | Representative illustration of the intermolecular interactions propagating in the crystal structure of compound FeB-HS (magenta dashed lines). The picture is quite similar for **FeB**-LS and is therefore not shown here. Magenta dots represent the centroid of the respective phenyl ring of the $[B(Ph)_4]^-$ anion and floating white dots are hydrogen atoms involved in intermolecular interactions. Hanging intermolecular bonds are drawn in green. Hydrogen atoms not involved in H-bonding were omitted for clarity.

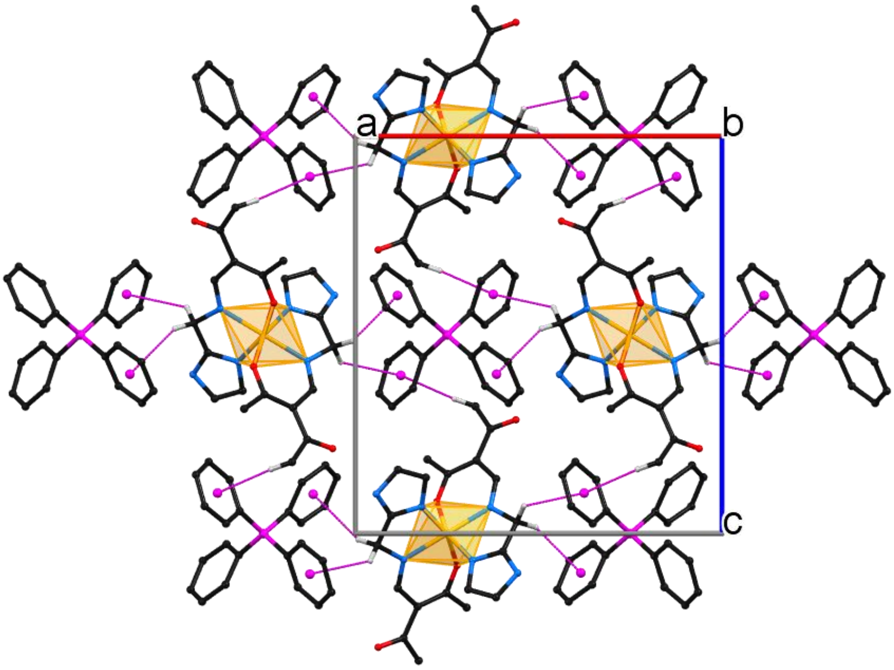

Supplementary Figure 13 | Representative illustration of the $CH_2\cdots\pi$ and $C_{Me}H\cdots\pi$ interactions propagating along the *ac* plane in the crystal structure of compound FeB-HS (magenta dashed lines). The picture is quite similar for **FeB**-LS and is therefore not shown here. Magenta dots represent the centroid of the respective phenyl ring of the $[B(Ph)_4]^-$ anion. Other hydrogen atoms were omitted for clarity.

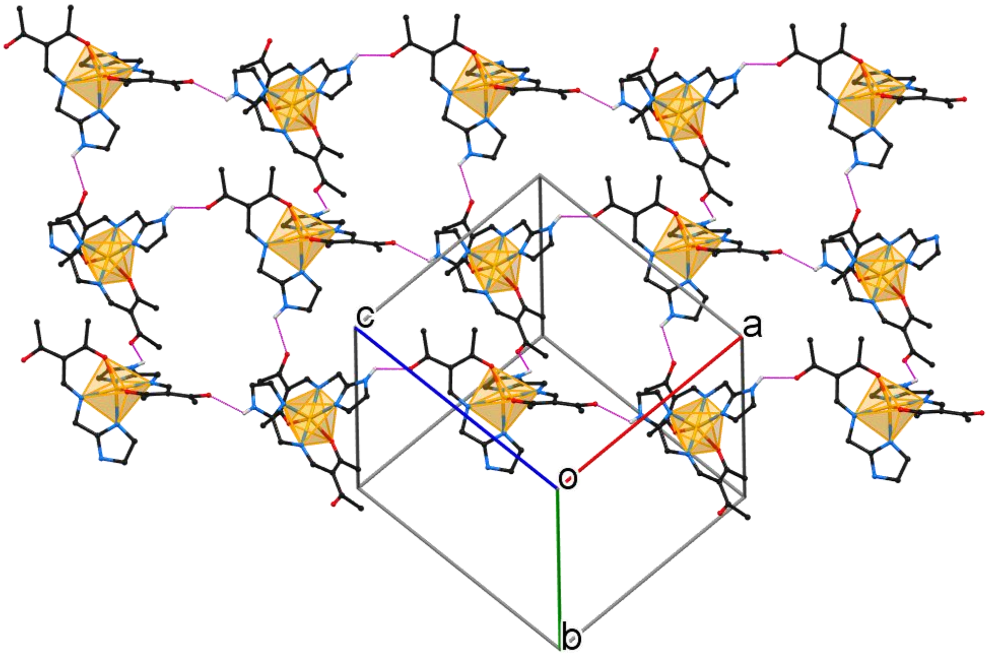

Supplementary Figure 14 | Representative illustration of the "classic" H-bonding network propagating along the *ac* plane in crystalline FeB-HS (magenta dashed lines). The picture is quite similar for **FeB**-LS and is therefore not shown here. Other hydrogen atoms as well as the $[B(Ph)_4]^-$ anions were omitted for clarity.

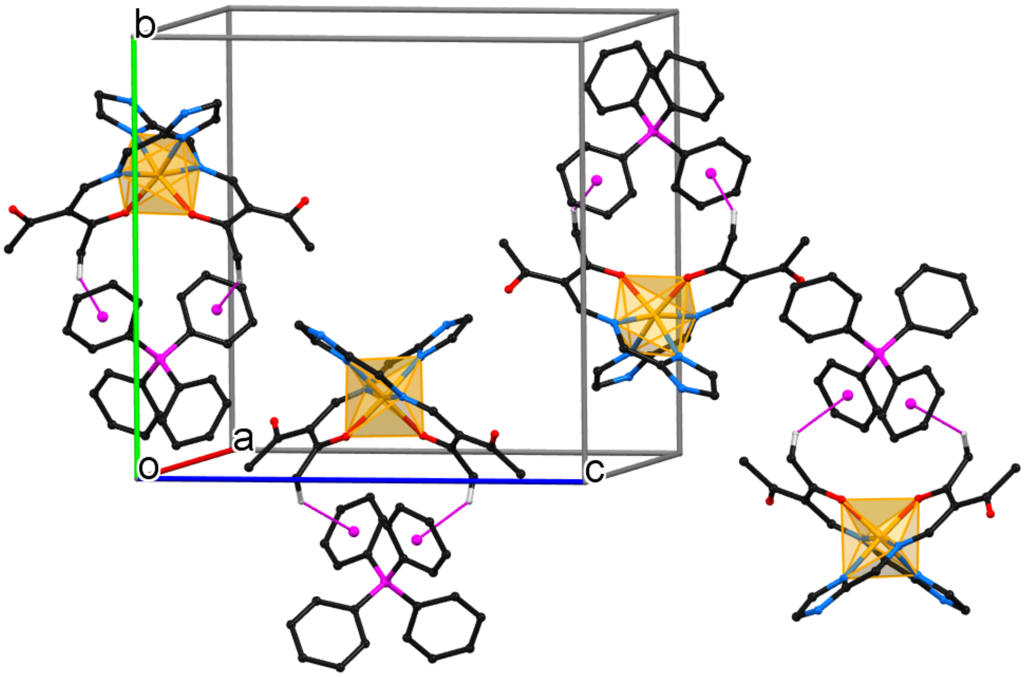

Supplementary Figure 15 | Representative illustration of the other $C_{Me}H\cdots\pi$ interactions connecting the individual *ac* layers in the crystal structure of compound FeB-HS (magenta dashed lines). The picture is quite similar for **FeB**-LS. Magenta dots represent the centroid of the respective phenyl ring of the $[B(Ph)_4]^-$ anion. Other hydrogen atoms were omitted for clarity.

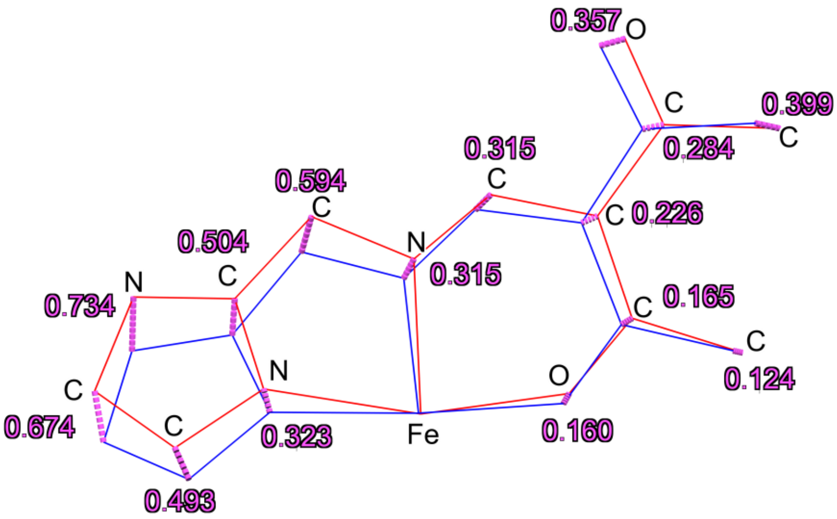

Supplementary Figure 16 | Structural overlay of the asymmetric unit of the complex cation in both different spin states (blue: LS state; red: HS state) in the wireframe model. The numbers indicate the atomic displacement upon spin transition (in Å) and demonstrate the size of structural rearrangement during spin state switch (in terms of ligand conformation).

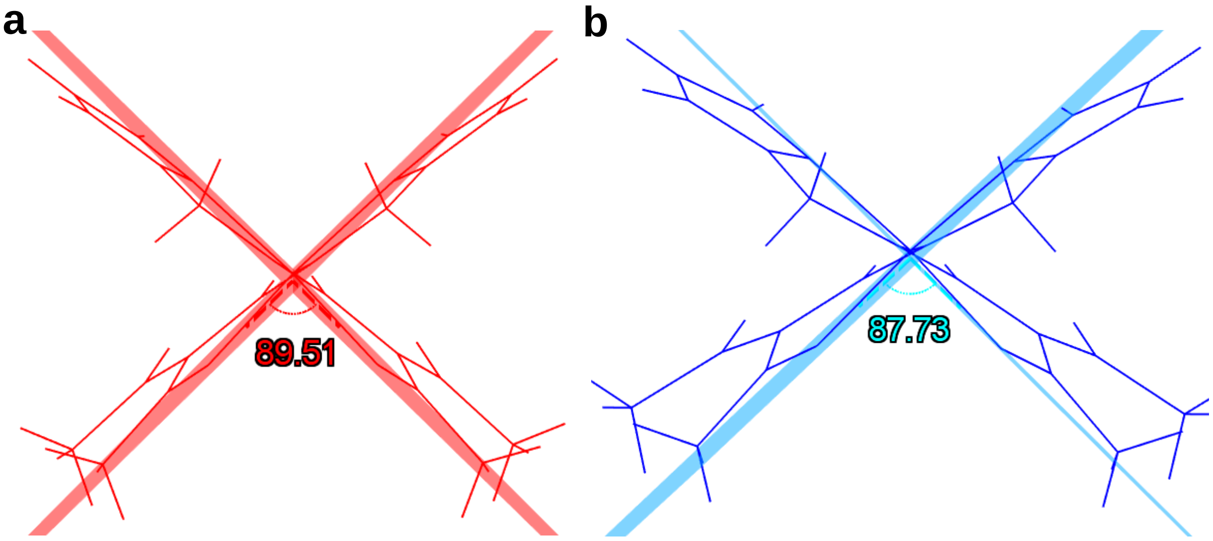

Supplementary Figure 17 | Molecular structure of the complex cations in the wireframe model. a HS state. **b** LS state. The numbers represent the value of θ in degree, a geometrical parameter introduced by Halcrow.[12,13] Usually, it would correspond to the dihedral angle between the least square planes of the two meridional-coordinating ligands. In this case θ is calculated by considering only the *NN'O* donor set ($\Delta\theta^{HS-LS} = 1.78°$).

3.5 Powder diffraction patterns of compound FeB

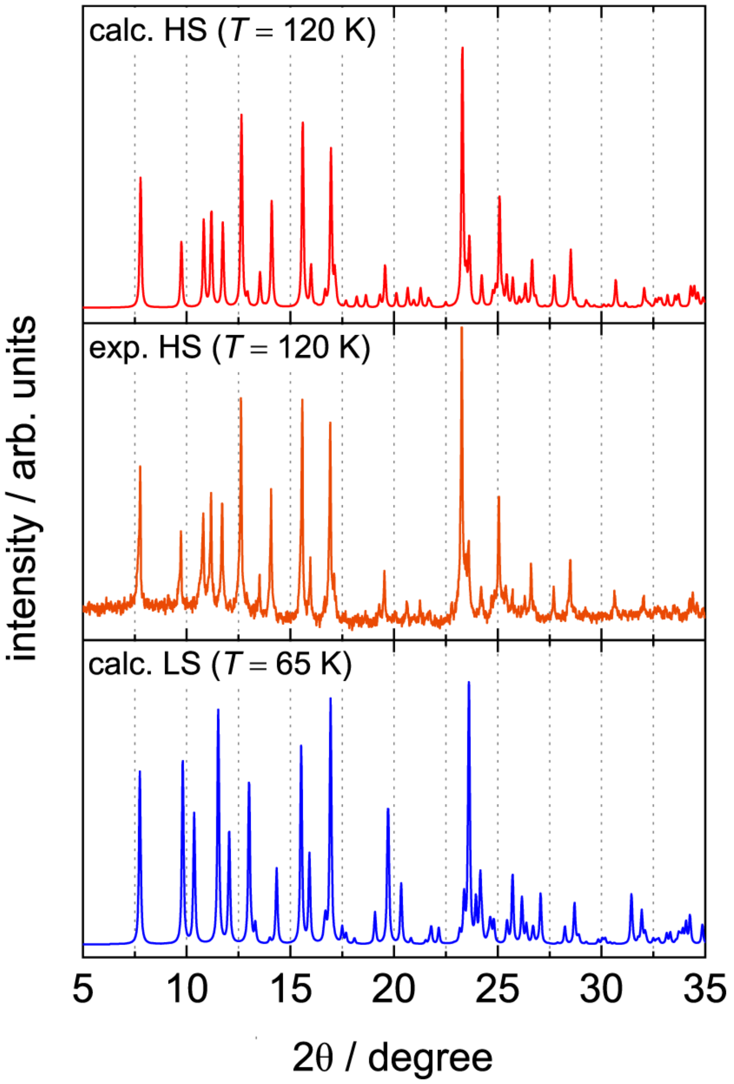

Supplementary Figure 18 | Experimental (*T* = 120 K) and simulated powder XRD patterns of compound FeB. The simulated patterns were calculated with Mercury[14] from single crystal diffraction data (*T* = 120, 65 K). A baseline correction was applied to the experimental data. The experimental data qualitatively matches the calculated diffraction pattern of **FeB**-HS which confirms the phase purity of compound **FeB**.

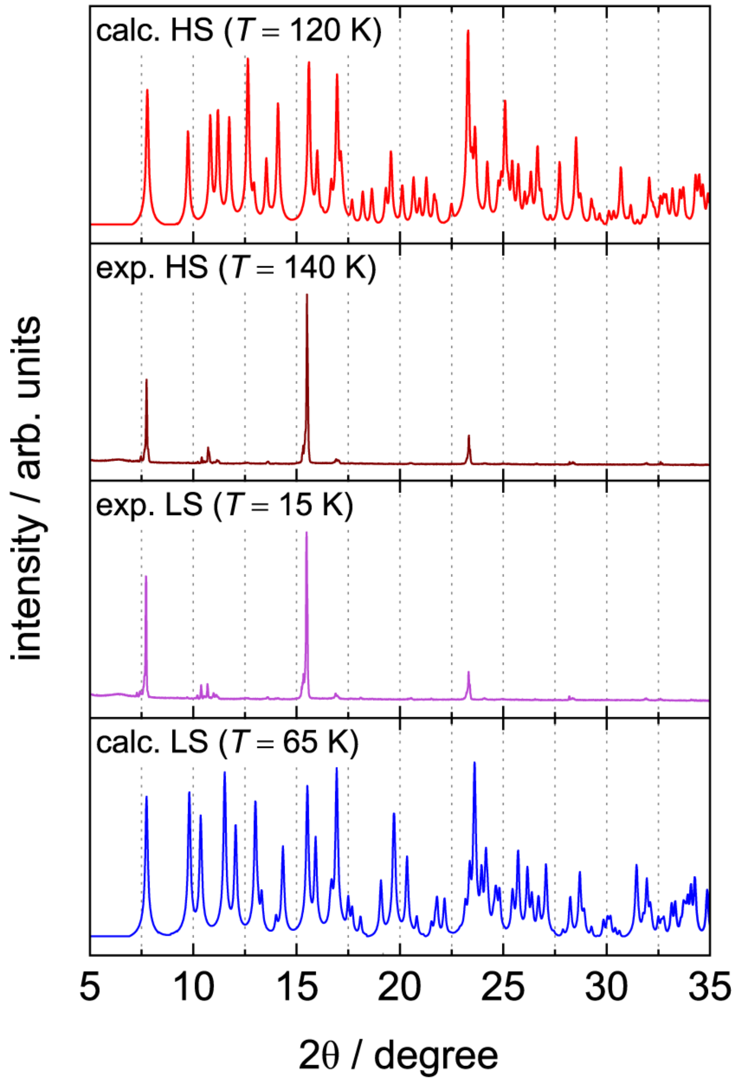

Supplementary Figure 19 | Experimental (T = 140, 15 K) and simulated powder XRD patterns of compound FeB. The simulated patterns were calculated with Mercury[14] from single crystal diffraction data (T = 120, 65 K). A baseline correction was applied to the experimental data. Although we have strong evidence that the complex molecules remain in the HS state after post-synthesis treatment (*e.g.*, grinding, necessary for well-resolved PXRD patterns), we still attempted to capture the diffraction pattern of the LS state. To avoid the use of ground material and thus maintaining the crystalline integrity required for the HS→LS transition, we placed as-synthesised crystallites of compound **FeB** onto the flat sample PXRD holder. In this way, random (isotropic) orientation cannot be guaranteed, resulting in a high degree of texturing (anisotropic effects). The preferred orientation of individual crystallites leads to the absence of several reflections, thus there is a systematic deviation from simulated patterns (*cf*. Supplementary Figure 18 where experimental and calculated data match due to sufficiently ground material). Mercury[14] can account for such preferred orientation to confirm that texturing is indeed the reason for the observations (not shown here). In agreement with the unchanged space group (*Pbcn*; crystallographic conservative spin transition), the diffraction patterns of **FeB**-HS and **FeB**-LS are almost unchanged.

4 SQUID MAGNETOMETRY

4.1 Scan rate dependent spin crossover

Supplementary Table 7 | Summary of SQUID (superconducting quantum interference device) experiments. The different runs were performed with three independently prepared samples. Real scan rates ($v_{\downarrow\uparrow}^{\text{real}}$) were determined by considering the number of data points and the actual time stamps written by the MPMS (magnetic property measurement system). Sweep mode (no internal temperature stabilisation) applies to all scan rates $v_{\downarrow\uparrow}$ if not stated otherwise. Small discrepancies among the individual series are due to imperfect crystals and different crystal sizes, inevitable crystal fracture during sample preparation and even weighing errors.

Scan rate $v_{\downarrow\uparrow}$	Run 1 [a, d]	$\chi_M T$	Run 2 [b, e]	$\chi_M T$	Run 3 [b]	$\chi_M T$	Run 4 [b]	$\chi_M T$	Run 5 [c]	$\chi_M T$
$v_{\downarrow\uparrow}^{\text{nominal}}$	$v_{\downarrow\uparrow}^{\text{real}}$	(T = 50 K)	$v_{\downarrow\uparrow}^{\text{real}}$	(T = 50 K)	$v_{\downarrow\uparrow}^{\text{real}}$	(T = 50 K)	$v_{\downarrow\uparrow}^{\text{real}}$	(T = 50 K)	$v_{\downarrow\uparrow}^{\text{real}}$	(T = 50 K)
10	↓5.20 ↑9.90	3.97	↓9.39 ↑9.90	4.41			↓5.40 ↑10.1	4.40	↓9.03 ↑9.69	4.25
8	↓3.95 ↑7.82	3.96	↓8.38 ↑7.82	4.39			↓3.76 ↑5.43	4.34		
5	↓3.75 ↑4.93	3.94	↓5.39 ↑4.93	4.32			↓3.33 ↑3.97	4.21	↓4.84 ↑4.93	3.72
4	↓3.43 ↑3.96	3.90	↓4.35 ↑3.96	4.20			↓2.97 ↑4.03	3.95		
3	↓2.78 ↑2.97	3.79	↓3.20 ↑2.98	3.96			↓2.48 ↑3.01	3.51		
2.5	↓2.54 ↑2.48	3.83								
2	↓1.97 ↑1.99	3.39			↓2.11 ↑1.85	3.43	↓1.96 ↑2.00	2.82	↓1.95 ↑2.00	1.69
1.8	↓1.82 ↑1.79	3.50								
1.5	↓1.53 ↑1.49	3.22								
1.3	↓1.34 ↑1.30	3.07								
1	↓1.02 ↑1.00	2.66	↓1.04 ↑0.96	2.40	↓1.05 ↑0.97	2.31	↓1.02 ↑1.00	1.77		
0.7	↓0.74 ↑0.70	2.29								
0.5	↓0.52 ↑0.50	2.00	↓0.49 ↑0.49	1.34			↓0.52 ↑0.50	1.22		
0.2	↓0.21 ↑0.20	1.32								
settle mode					↓0.36 ↑0.42 [a]	1.34			↓0.91 ↑1.39	1.00
K min^{-1}	K min^{-1}	cm^3 K mol^{-1}	K min^{-1}	cm^3 K mol^{-1}	K min^{-1}	cm^3 K mol^{-1}	K min^{-1}	cm^3 K mol^{-1}	K min^{-1}	cm^3 K mol^{-1}

Supplementary Table 7, cont.

[a] 12.17 mg, batch #1.

[b] 6.10 mg, batch #2.

[c] 13.86 mg, batch #3.

[d] Run 1 was interrupted due to technical reasons. This required removal and re-centering of the sample with respect to the SQUID coils. Thus, the rates $v\downarrow = 2.5$, 1.8, 1.5, 1.3, 0.7, and 0.2 K min^{-1} may appear as if they systematically do not match the other cooling velocities.

[e] This run also included the TIESST experiment. The same outcome (*i.e.*, T_{TIESST}) was obtained with 1.89 mg of batch #2.

Note (#1): After each cooling-heating cycle the very same sample was maintained at 400 K for 1 h to ensure a "thermal reset". It is emphasized that independently synthesized batches of compound **FeB** exhibit excellent consistency between the individual series, and the same results are obtained even on older samples (>1 year).

Note (#2): Data can only be reliably reproduced if independently synthesised samples are treated in the same way during SQUID sample preparation. Preservation of crystalline integrity is very important. Post treatment, *e.g.*, grinding will ultimately lead to a decrease in cooperativity. This is certainly associated with the importance and the extension of the network of intermolecular interactions.

4.1.1 Run 1

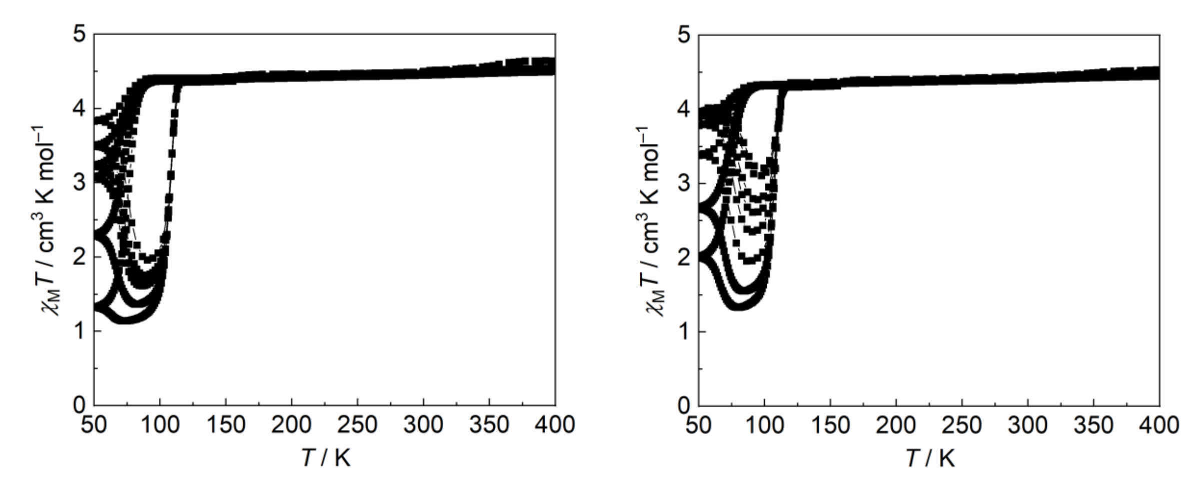

Supplementary Figure 20 | Plot of $\chi_M T$ *vs.* T over the entire temperature range T = 400–50 K with different scan rates $v{\downarrow\uparrow}$ for compound **FeB**. Run 1 consists of two individual series of the same sample. Line is guidance for the eye. The explicit specification of respective scan rates is given in Supplementary Figure 21.

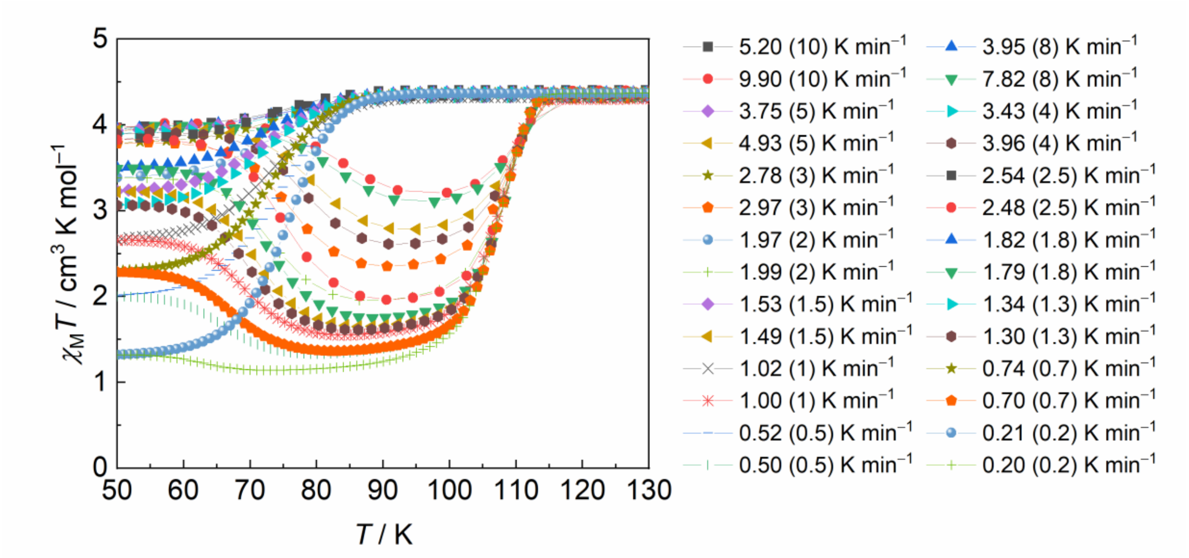

Supplementary Figure 21 | Plot of $\chi_M T$ *vs.* T between T = 130–50 K with different scan rates $v{\downarrow\uparrow}^{real}$ for compound **FeB** in run 1. Line is guidance for the eye. Nominal scan rates $v{\downarrow\uparrow}$ are quoted in brackets.

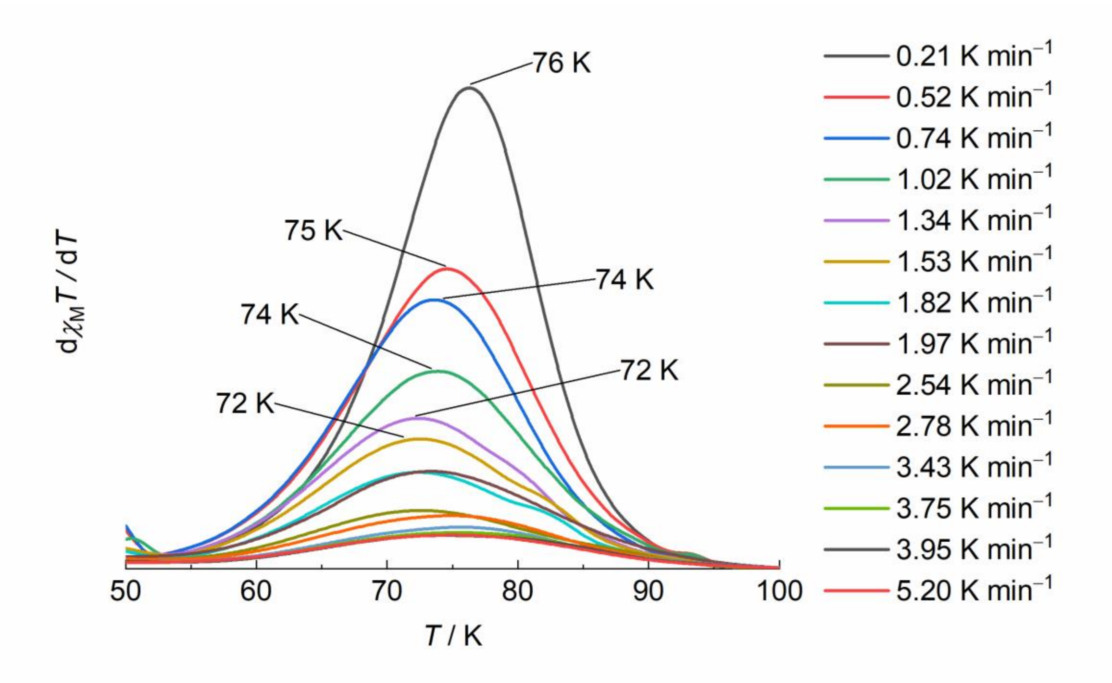

Supplementary Figure 22 | First derivatives of $\chi_M T$ data in the cooling branch/cycle at various cooling rates $v\downarrow$ in run 1. The respective maximum corresponds to the transition temperature $T_{½}$. Curves are illustrated as a B-spline function.

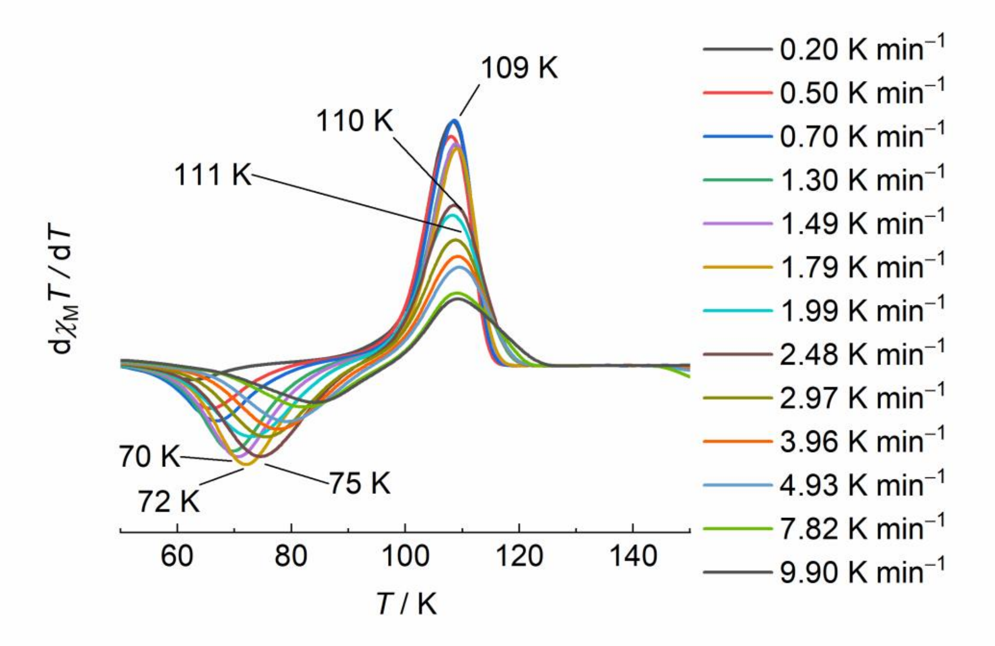

Supplementary Figure 23 | First derivatives of $\chi_M T$ data in the heating branch/cycle at various cooling rates $v\downarrow$ in run 1. The respective maximum/minimum corresponds to the transition temperature $T_{½}$. Curves are illustrated as a B-spline function.

4.1.2 Run 2

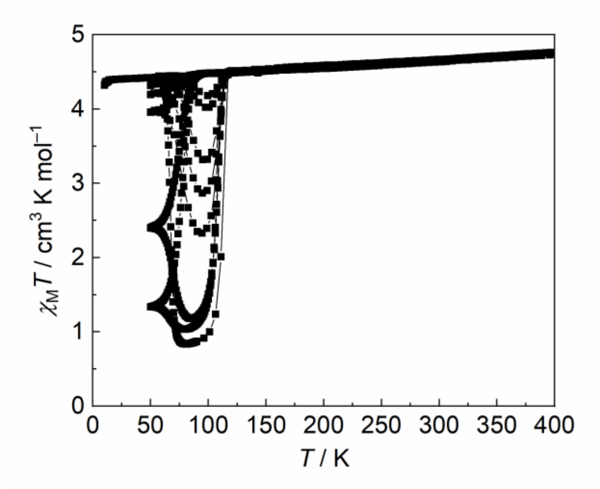

Supplementary Figure 24 | Plot of $\chi_M T$ *vs.* T over the entire temperature range T = 400–2 K with different scan rates v↓↑ for compound **FeB** including the TIESST experiment in run 2. Line is guidance for the eye. The explicit specification of respective scan rates is given in Supplementary Figure 25.

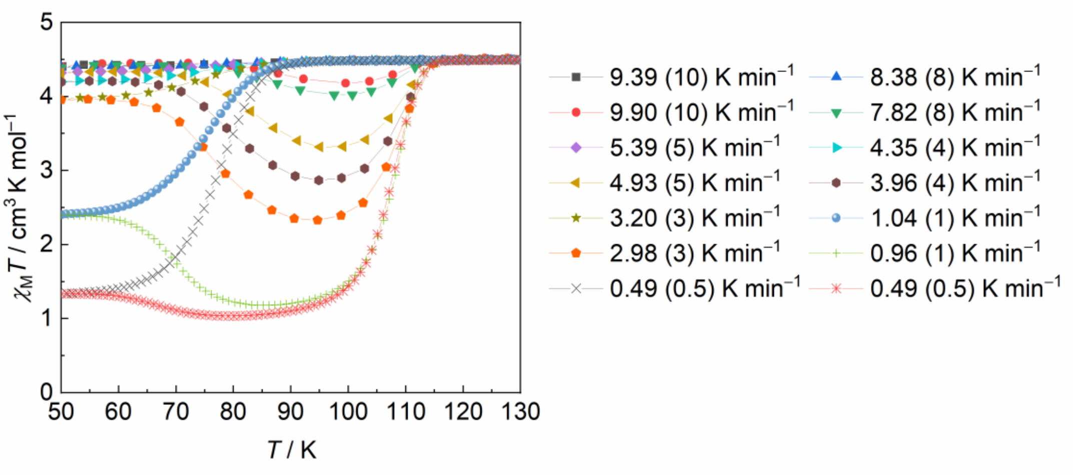

Supplementary Figure 25 | Plot of $\chi_M T$ *vs.* T between T = 130–50 K with different scan rates v↓↑^{real} for compound **FeB** in run 2. Line is guidance for the eye. Nominal scan rates v↓↑ are quoted in brackets.

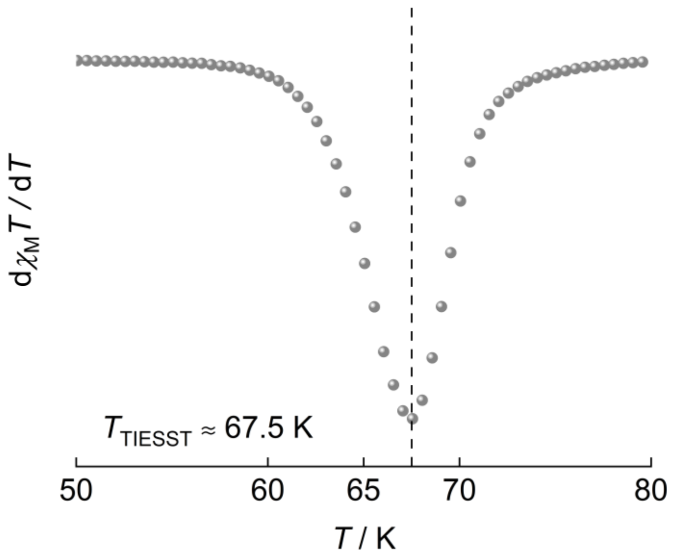

Supplementary Figure 26 | First derivative of $\chi_M T$ data in the TIESST experiment at $v\uparrow$ = 0.3 K min^{-1} heating rate. The respective minimum corresponds to T_{TIESST}.

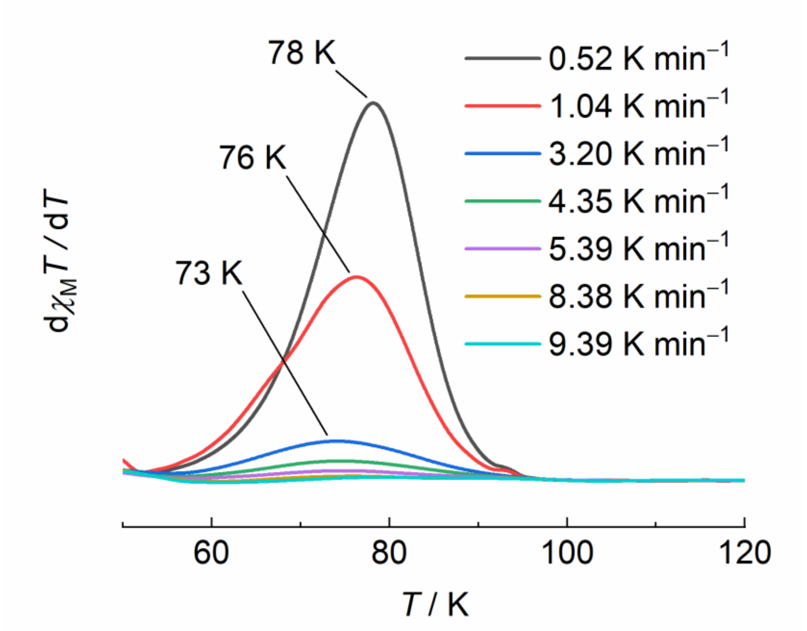

Supplementary Figure 27 | First derivatives of $\chi_M T$ data in the cooling branch/cycle at various cooling rates $v\downarrow$ in run 2. The respective maximum corresponds to the transition temperature $T_{½}$. Curves are illustrated as a B-spline function.

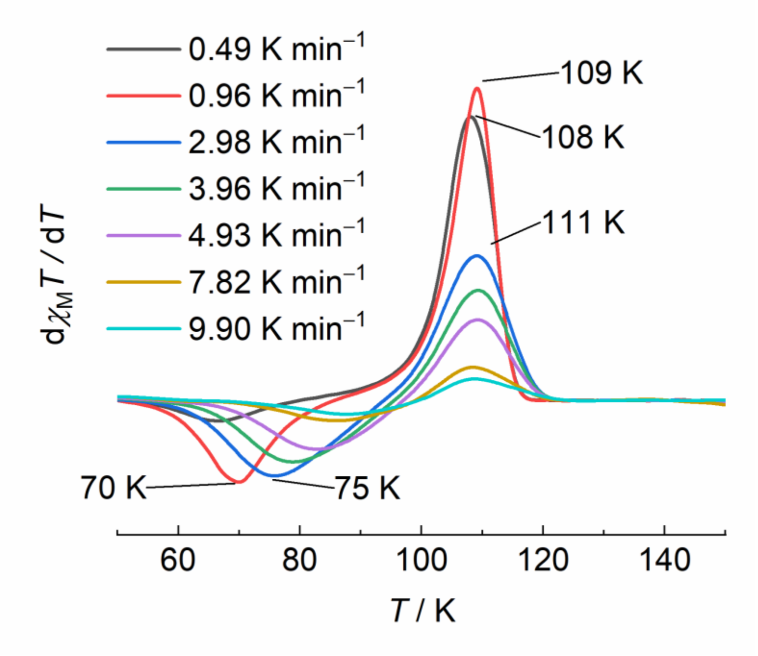

Supplementary Figure 28 | First derivatives of $\chi_M T$ data in the heating branch/cycle at various cooling rates $v_\downarrow$ in run 2. The respective maximum/minimum corresponds to the transition temperature $T_{½}$. Curves are illustrated as a B-spline function.

4.1.3 Run 3

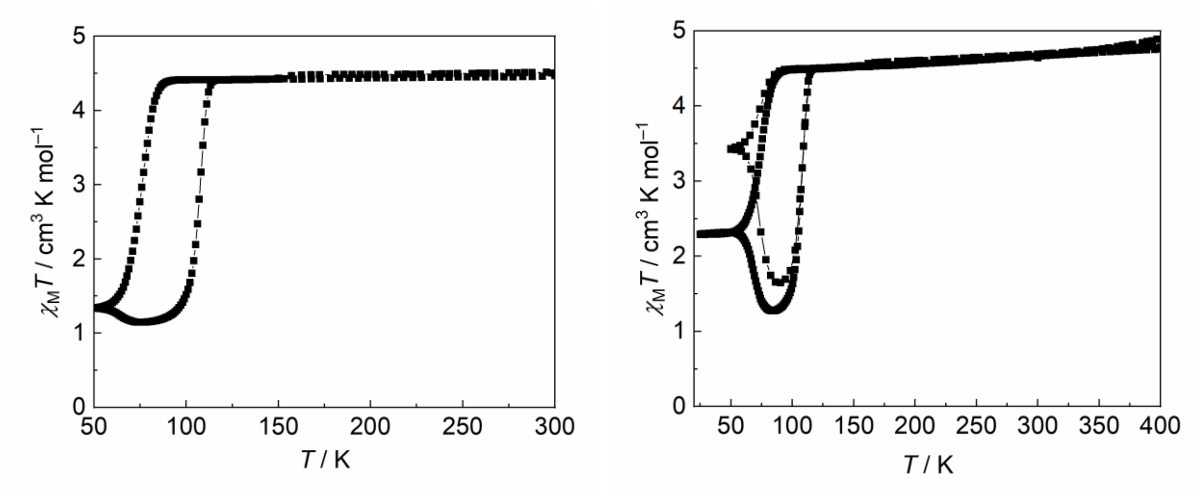

Supplementary Figure 29 | Plot of $\chi_M T$ *vs.* T over the entire temperature range $T = 400–50$ K with different scan rates $v↓↑$ for compound **FeB**. Run 3 consists of two individual series of the same sample. Line is guidance for the eye. The explicit specification of respective scan rates is given in Supplementary Figure 30.

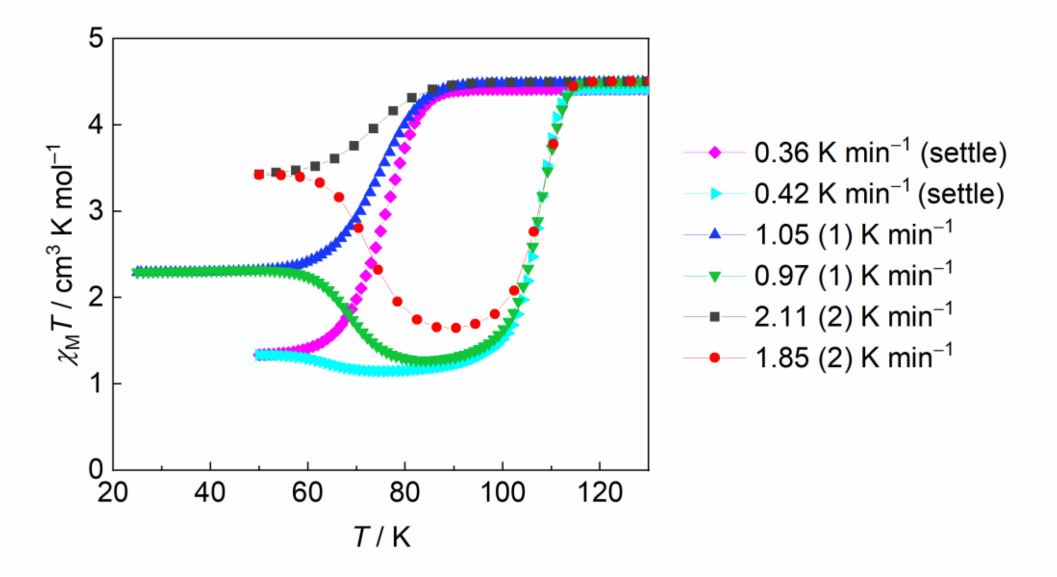

Supplementary Figure 30 | Plot of $\chi_M T$ *vs.* T between $T = 130–50$ K with different scan rates $v↓↑^{real}$ for compound **FeB** in run 3. Line is guidance for the eye. Nominal scan rates are quoted in brackets.

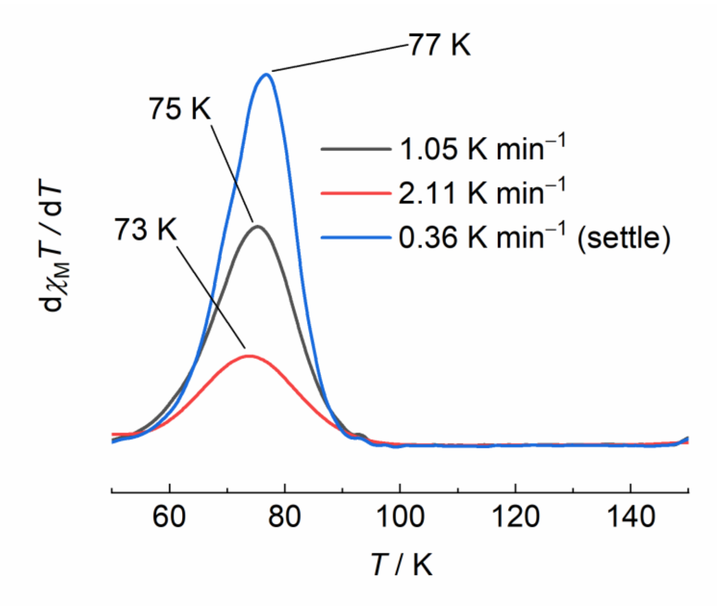

Supplementary Figure 31 | First derivatives of $\chi_M T$ data in the cooling branch/cycle at various cooling rates v↓ in run 3. The respective maximum corresponds to the transition temperature $T_{½}$. Curves are illustrated as a B-spline function.

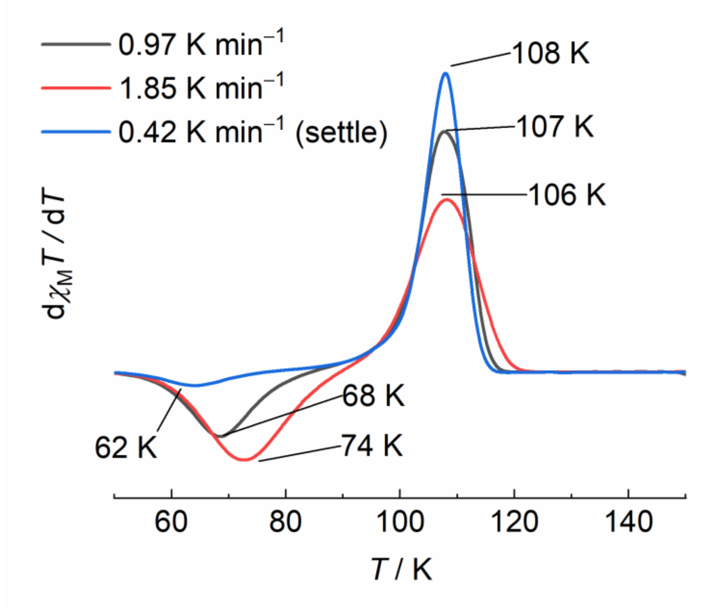

Supplementary Figure 32 | First derivatives of $\chi_M T$ data in the heating branch/cycle at various cooling rates v↓ in run 3. The respective maximum/minimum corresponds to the transition temperature $T_{½}$. Curves are illustrated as a B-spline function.

4.1.4 Run 4

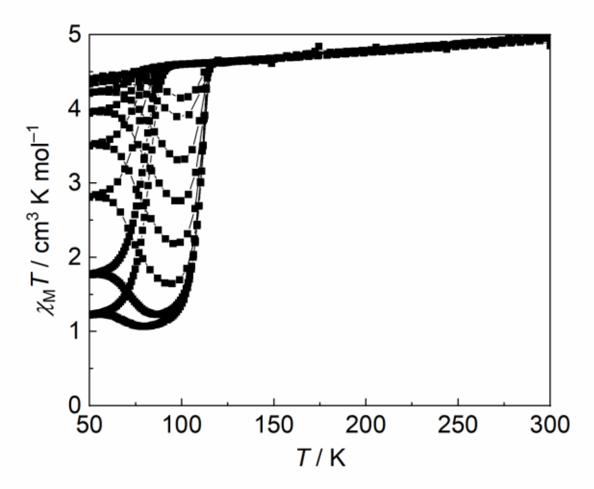

Supplementary Figure 33 | Plot of $\chi_M T$ *vs.* T over the entire temperature range T = 300–50 K with different scan rates $v_{\downarrow\uparrow}$ for compound **FeB** in run 4. Line is guidance for the eye. The explicit specification of respective scan rates is given in Supplementary Figure 34.

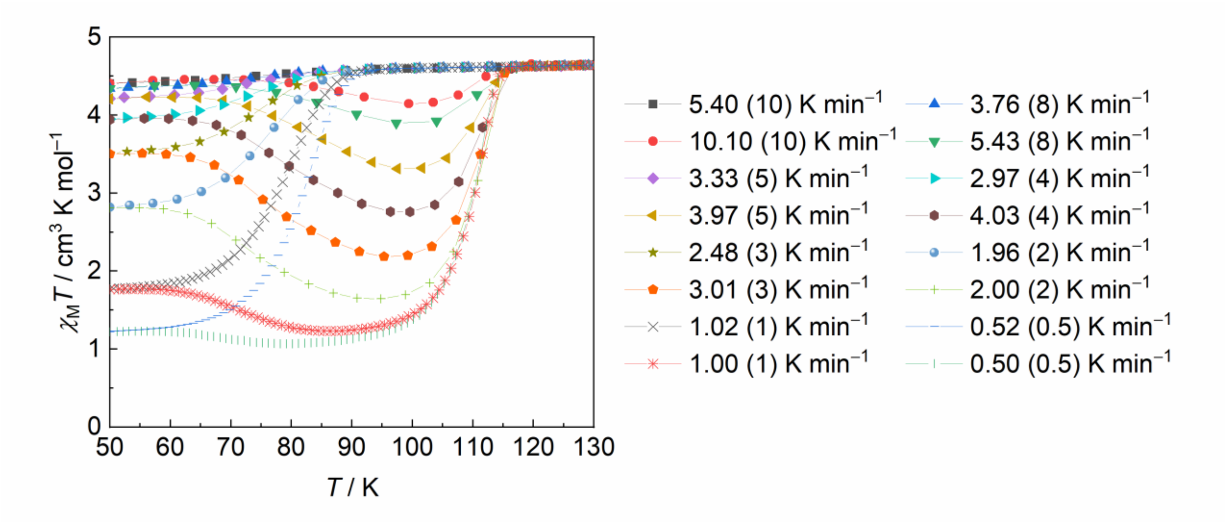

Supplementary Figure 34 | Plot of $\chi_M T$ *vs.* T between 130–50 K with different scan rates $v_{\downarrow\uparrow}^{real}$ for compound **FeB** in run 4. Line is guidance for the eye. Nominal scan rates are quoted in brackets.

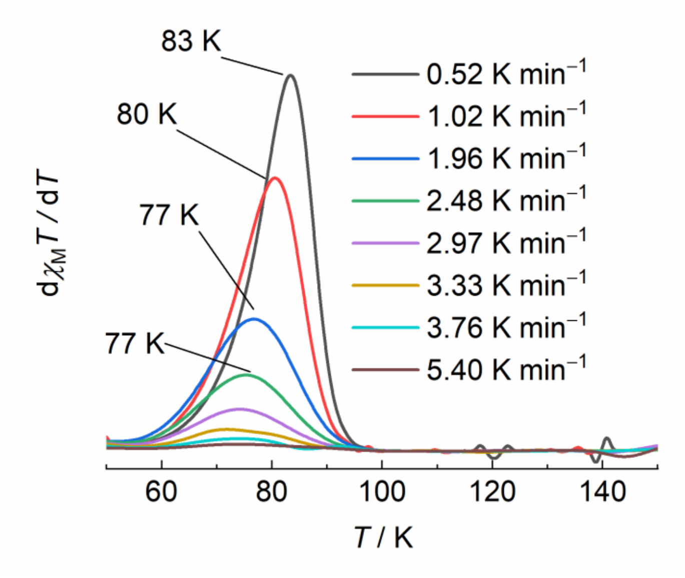

Supplementary Figure 35 | First derivatives of the $\chi_M T$ data in the cooling branch/cycle at various cooling rates $v\downarrow$ in run 4. The respective maximum corresponds to the transition temperature $T_{½}$. Curves are illustrated as a B-spline function.

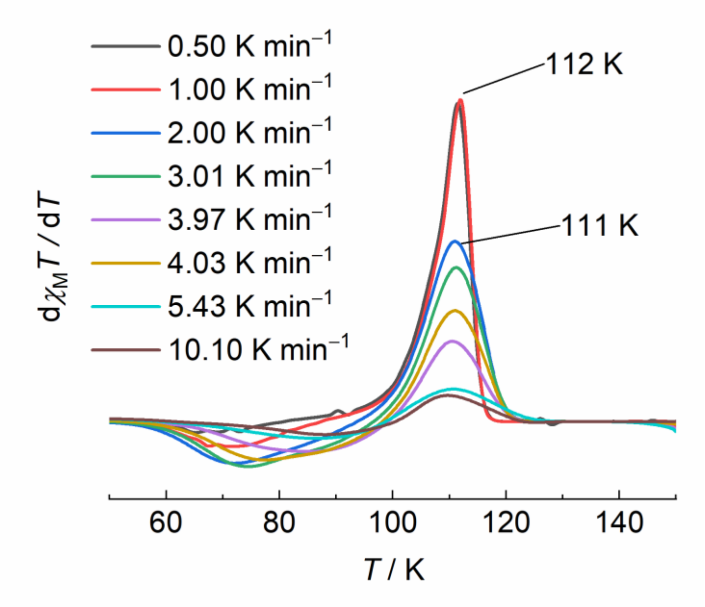

Supplementary Figure 36 | First derivatives of the $\chi_M T$ data in the heating branch/cycle at various cooling rates $v\downarrow$ in run 4. The respective maximum/minimum corresponds to the transition temperature $T_{½}$. Curves are illustrated as a B-spline function.

4.1.5 Run 5

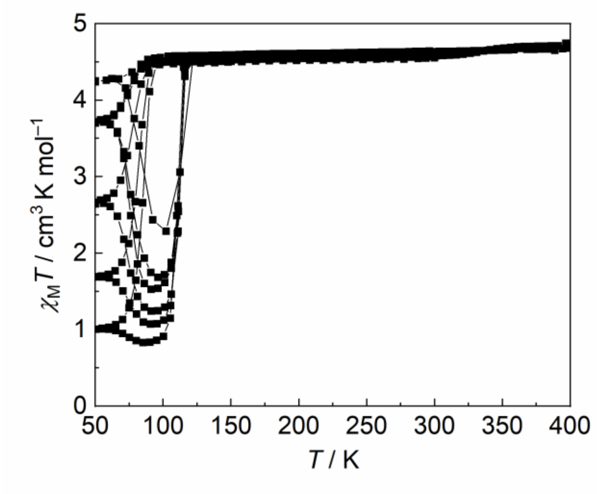

Supplementary Figure 37 | Plot of $\chi_M T$ *vs.* T over the entire temperature range T = 300–50 K with different scan rates $v_{\downarrow\uparrow}$ for compound **FeB** in run 5. Line is guidance for the eye. The explicit specification of respective scan rates is given in Supplementary Figure 38.

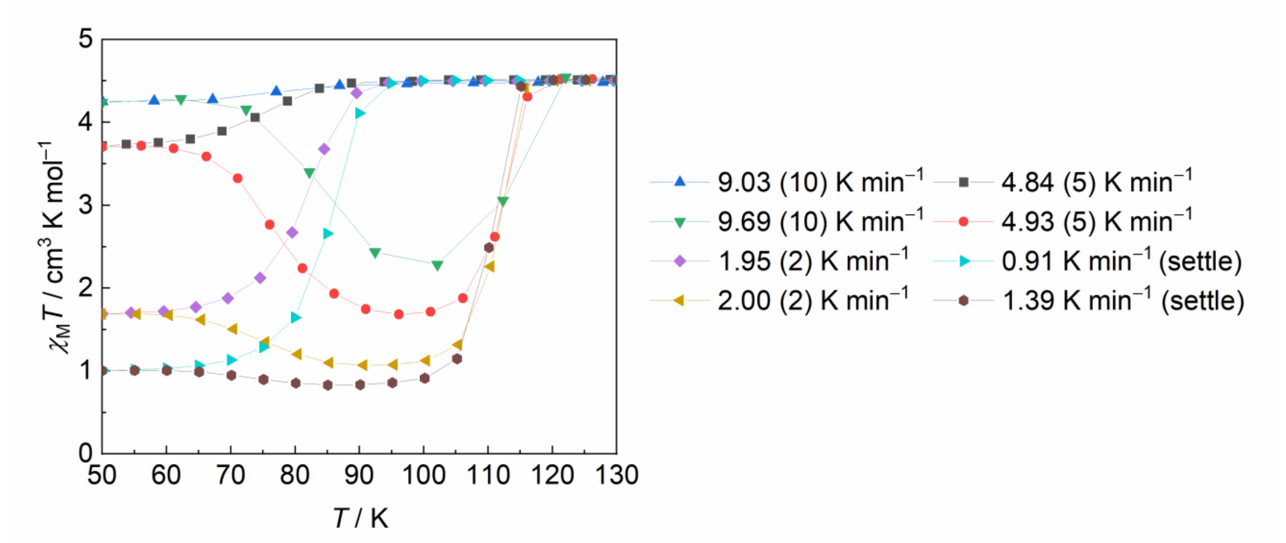

Supplementary Figure 38 | Plot of $\chi_M T$ *vs.* T between T = 130–50 K with different scan rates $v_{\downarrow\uparrow}^{real}$ for compound **FeB** in run 5. Line is guidance for the eye. Nominal scan rates are quoted in brackets.

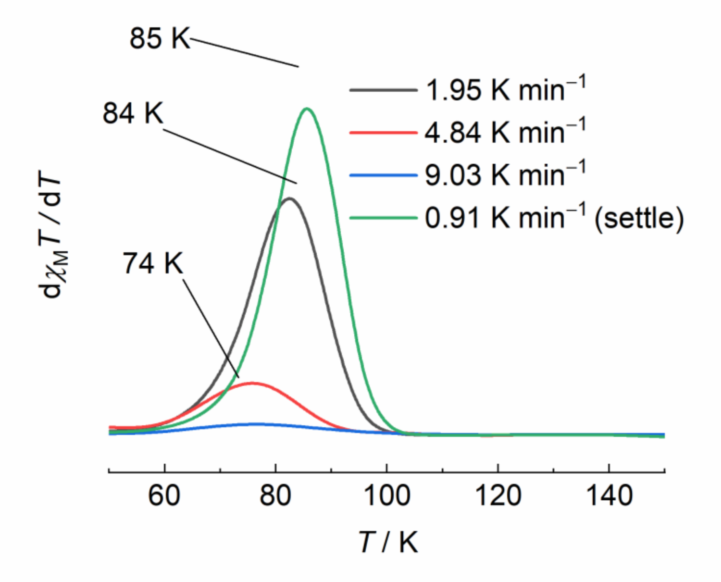

Supplementary Figure 39 | First derivatives of $\chi_M T$ data in the cooling branch/cycle at various cooling rates $v_\downarrow$ in run 5. The respective maximum corresponds to the transition temperature $T_{½}$. Curves are illustrated as a B-spline function.

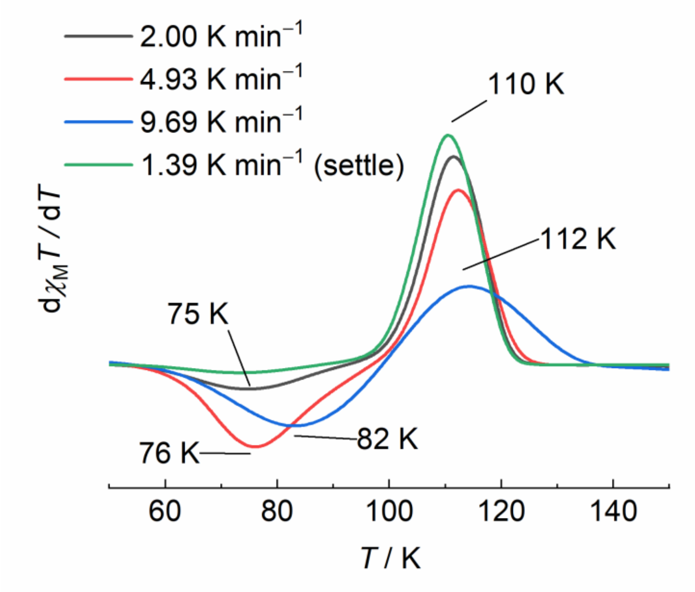

Supplementary Figure 40 | First derivatives of $\chi_M T$ data in the heating branch/cycle at various cooling rates $v_\downarrow$ in run 5. The respective maximum/minimum corresponds to the transition temperature $T_{½}$. Curves are illustrated as a B-spline function.

Supplementary Table 8 | Selected magnetic data of compound **FeB** from different runs (*cf*. Figure 6 and 7 in the manuscript).

Nominal scan rate v↓↑	$\chi_M T$ (T = 50 K)	$\chi_M T$ (minimum) [a]	$T_{½}$ [b]	Hysteresis width
10	4.40	4.18	n.a.	n.a.
8	4.39	4.02	n.a.	n.a.
5	4.32	3.31	n.a.	n.a.
4	4.20	2.86	↓73 ↑111	38
3	3.96	2.33	↓73 ↑111	38
2	3.43	1.65	↓74 ↑106	32
1	2.32	1.27	↓76 ↑109	33
0.5	1.33	1.15	↓78 ↑108	30
Settle mode	1.00	1.04	↓77 ↑108	31
Quasi-static hysteresis	n.a	n.a	n.a.	30 [c]
Hidden hysteresis	n.a	n.a	n.a.	43 [d]
K min^{-1}	cm^3 K mol^{-1}	cm^3 K mol^{-1}	K	K

[a] Lowest $\chi_M T$ values were taken from the heating branch.
[b] Transition temperatures $T_{½}$ were obtained from the respective first derivatives.
[c] Quasi-static hysteresis was estimated from the isothermal relaxation data.
[d] Hidden hysteresis was estimated from the TIESST-related relaxation curve and heating branch.

4.2 Relaxation dynamics

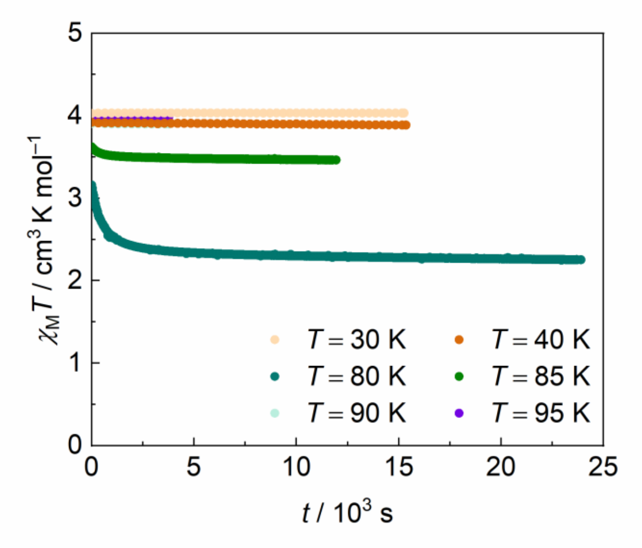

Supplementary Figure 41 | Isothermal dynamics of compound **FeB** as a plot of $\chi_M T$ *vs*. t at T = 30, 40, 80, 85, 90, 95 K. Please note the overlap of the corresponding graphs at T = 30, 40, 90, 95 K, clearly indicating the existence of the $HS_{(TR)}$ state at these temperatures. All the temperatures shown here and in Supplementary Figure 42 were adjusted by fast cooling. The MPMS system has a finite time demand of temperature stabilisation, therefore some values of $\chi_M T(t = 0)$ are already lower than would be expected due to fast HS⟶LS relaxation.

Supplementary Table 9 | Data extracted from isothermal relaxations for Arrhenius plot. The transition of the metastable HS state to the LS state is a highly cooperativity-driven process in compound **FeB**, evidenced by the strong deviation of the relaxation curves from single-exponential (as would be expected for systems with diluted SCO-active centres). The curves rather reflect a sigmoidal shape which arises from the self-accelerating process of the SCO.[15,16]

T	k_{HL} (slope) [a]	error	T^{-1}	ln k_{HL}	error [b]	R^2
50	−6.586E−6	4.54E−8	0.02	−11.93056	−0.00689	0.92557
52	−1.417E−5	2.464E−7	0.01923	−11.16438	−0.01739	0.98247
54	−4.239E−5	8.407E−7	0.01852	−10.0686	−0.01983	0.98987
56	−1.025E−4	2.816E−7	0.01786	−9.18565	−0.00275	0.98512
58	−1.525E−4	2.883E−6	0.01724	−8.78835	−0.0189	0.99862
60	−3.32E−4	7.706E−6	0.01667	−8.01038	−0.02321	0.99892
65	−0.00212	4.78E−5	0.01538	−6.15634	−0.02255	0.99898
70	−0.00321	2.15334E−5	0.01429	−5.74148	−0.00671	0.99991
75	−0.00231	4.1554E−5	0.01333	−6.07051	−0.01799	0.99935
30	n.a.	n.a.	0.03333	n.a.	n.a.	n.a.
40	n.a.	n.a.	0.025	n.a.	n.a.	n.a.
K	s^{-1}		K^{-1}			

[a] Rate constants k_{HL} were determined by estimating the regime where the onset of the HS⟶LS relaxation appears to be linear (*cf*. logarithmic plot in Supplementary Figure 42a).

[b] Error is derived from linear propagation of uncertainty.

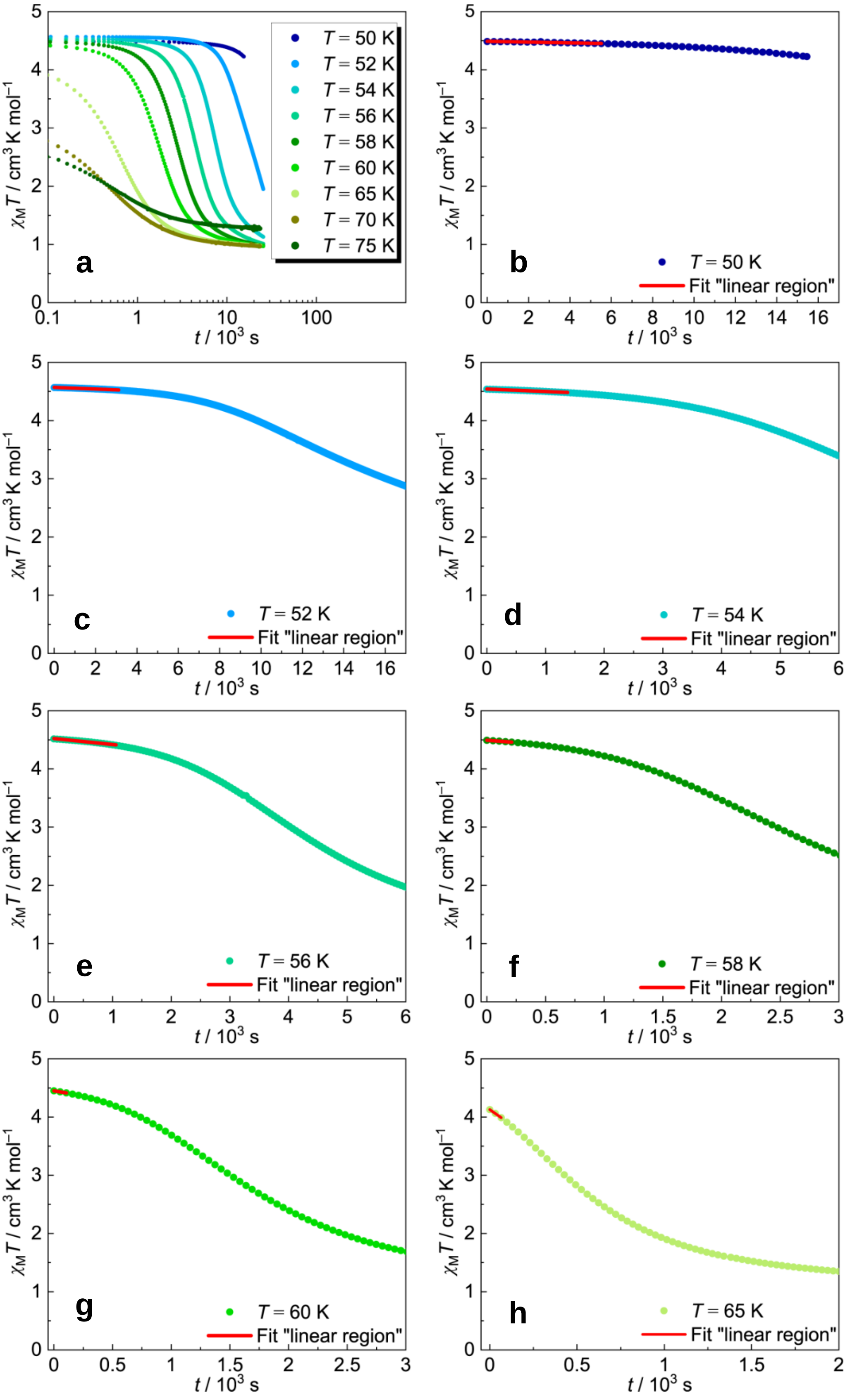

Supplementary Figure 42 | Isothermal dynamics plotted as $\chi_M T$ *vs.* t for compound FeB. a Logarithmic-scaled time-dependent evolution of $HS_{(TR)}$→LS relaxation at constant temperatures. **b–h** Time-dependent evolution of the $HS_{(TR)}$→LS relaxation T = 50, 52, 54, 56, 58, 60, 65, 70, 75 K and linear fitting data for $k_{HL}(T)$, drawn in red.

5 ^{57}FE MÖSSBAUER SPECTROSCOPY

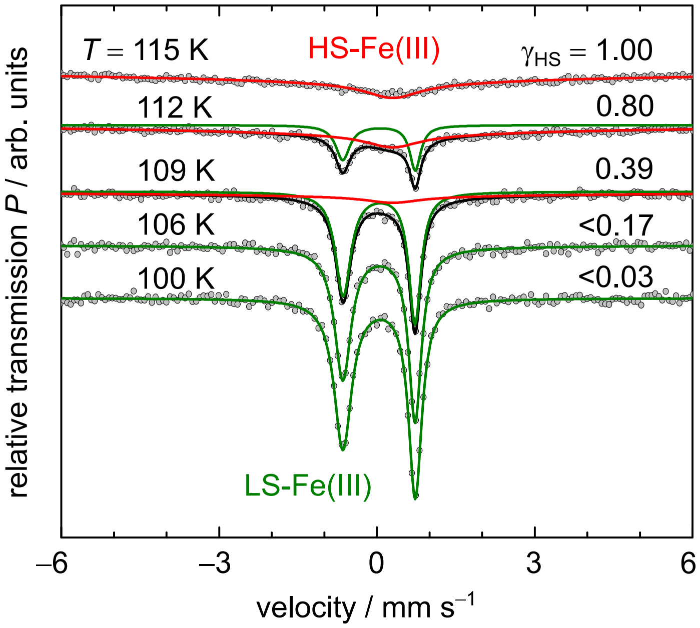

Supplementary Figure 43 | Zero-field Mössbauer spectra of compound **FeB**, recorded at $T = 100$, 106, 109, 112 and 115 K after a sequence of temperature steps: *i.e.*, (*i*) fast cooling to $T = 4$ K (with a cooling rate $v_{\downarrow} >$ 10 K min^{-1}), (*ii*) warming to $T = 90$ K, (*iii*) cooling back to $T = 4$ K. The spectrum of the relaxed LS state, after this sequence, at $T = 4$ K is shown in Supplementary Figure 50. Additional spectra (not shown) were recorded after subsequent warming to $T = 20$, 40, 60 and 80 K (*cf*. Supplementary Table 10). Symbols: Experimental data. Lines: Fit with the Blume-Tjon relaxation model.[17] The parameters of the fit at a given temperature are summarised in Supplementary Table 10. The displayed data recorded at $T = 100$ and 106 K were fitted with a single-site analysis (γ_{HS} was estimated at these temperatures in a second step with an independently performed two-component fit, accounting for a possible HS fraction and by assuming that the parameters of the HS fraction are similar to those obtained at $T = 150$ K; *cf*. Supplementary Table 10 and Supplementary Table 12). The displayed data recorded at $T = 109$ and 112 K were analysed with two components, which are attributed to the LS (green) and HS states (red) of compound FeB, reflecting the SCO transition at these temperatures. The black lines represent the superposition of the given LS and HS subspectra. The spectrum at $T = 115$ K shows no contribution of a LS state and was therefore fitted with a single ^{57}Fe site.

Supplementary Table 10 | Zero-field Mössbauer parameters of compound **FeB**, determined with a fit based on the Blume-Tjon relaxation model.[17] The isomer shift δ is specified relative to metallic iron at room temperature but was not corrected for the second-order Doppler shift. Γ denotes the half (Lorentzian) line widths at half maximum (HWHM), ΔE_Q the quadrupole splitting and ν_c the fluctuation rate of the local magnetic hyperfine field B_{hf} of the components (subspectra) used in the fit. The parameters δ, ΔE_Q, B_{hf}, Γ_{HWHM}, ν_c (LS) and $\nu_c B_{hf}^{-1}$ (LS) were determined with a single-site analysis; the values of γ_{HS} were separately determined with a two-component model. The parameters of the HS subspectrum were taken from the $T = 150$ K measurement (Supplementary Table 12) and were fixed in the fit (*cf.* values at $T = 115$ or 125 K in the present Supplementary Table 10).

T	δ	ΔE_Q	B_{hf}	Γ_{HWHM}	ν_c (LS)	$\nu_c B_{hf}^{-1}$ (LS)	γ_{HS} [b]	ν_c (HS)
20 [a]	0.158(14)	−1.48(3)	18.6*	0.188(11)	10.1(8)	0.54(4)	<12	5.9*
40	0.166(23)	−1.46(4)	18.6*	0.210(14)	7.1(6)	0.38(3)	<9	5.9*
60	0.169(11)	−1.41(2)	18.6*	0.165(9)	13(1)	0.70(5)	<10	5.9*
80	0.169(5)	−1.38(1)	18.6*	0.142(6)	33(3)	1.77(16)	<1	5.9*
100	0.162(4)	−1.37(1)	18.6*	0.140(6)	72(11)	3.87(59)	<3	5.9*
103	0.163(3)	−1.36(1)	18.6*	0.137(4)	73(8)	3.92(43)	<13	5.9*
106	0.161(4)	−1.37(1)	18.6*	0.139(6)	74(12)	3.98(65)	<17	5.9*
109	0.163(4)	−1.36(1)	18.6*	0.128(7)	95(18)	5.11(97)	39	5(2)
112	0.161(8)	−1.37(2)	18.6*	0.115(14)	95(39)	5.11(2.10)	80	6(1)
115	0.55*	0.26*	51*	0.48*	-	-	100	5.7(6)
125	0.55*	0.26*	51*	0.48*	-	-	100	5.7(7)
K	mm s^{-1}	mm s^{-1}	T	mm s^{-1}	mm s^{-1}	mm s^{-1} T^{-1}	%	mm s^{-1}

[a] Recorded after a sequence of temperature steps: *i.e.*, (*i*) fast cooling to $T = 4$ K, (*ii*) warming to $T = 90$ K and (*iii*) cooling back to $T = 4$ K.

[b] Values of γ_{HS} at $T \leq 106$ K are estimated upper limits, reflecting a large methodical uncertainty caused by the strong relaxation broadening associated with the HS state.

*) Fixed in the fit.

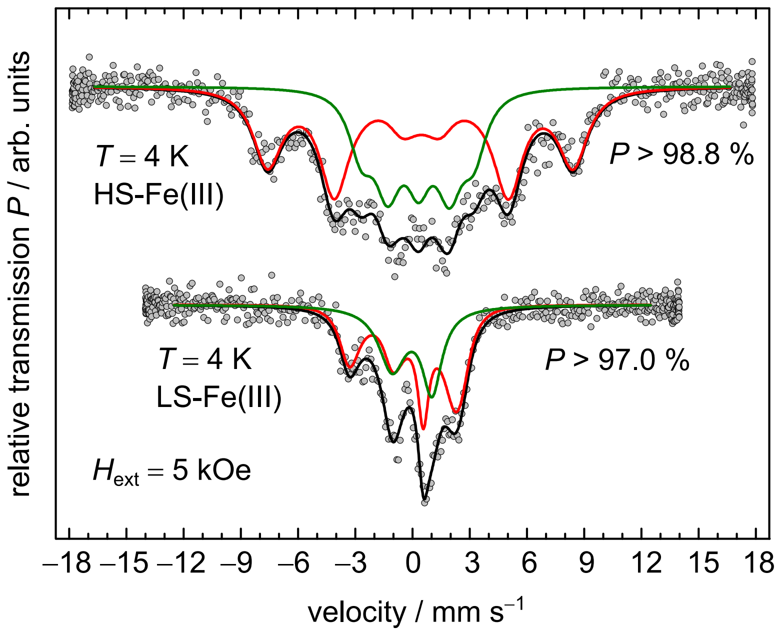

Supplementary Figure 44 | Mössbauer spectra of compound **FeB**, recorded at $T = 4$ K with an applied magnetic field of H_{ext} = 5 kOe. The upper spectrum was recorded after fast (field-)cooling (with a cooling rate v↓ > 10 K min^{-1}). The lower spectrum was recorded after a sequence of temperature steps: *i.e.*, (*i*) fast (field-)cooling to $T = 4$ K, (*ii*) (field-) warming to $T = 90$ K and (*iii*) (field-)cooling back to $T = 4$ K. Symbols: Experimental data. Lines: Fit with the Blume-Tjon relaxation model.[17] The parameters of the fit are summarised in Supplementary Table 11. For both measurements, two components (red and green) were used in the fit to account for the data. The black line represents the superposition of these two subspectra, respectively.

Supplementary Table 11 | Mössbauer parameters of the measurements on compound **FeB** with an applied magnetic field of H_{ext} = 5 kOe. The given parameters were determined with a fit based on the Blume-Tjon relaxation model.[17] The isomer shift δ is specified relative to metallic iron at room temperature but was not corrected for the second-order Doppler shift. Γ denotes the half (Lorentzian) line widths at half maximum (HWHM), ΔE_Q the quadrupole splitting, ν_c the fluctuation rate of the local magnetic hyperfine field B_{hf} and V the volume fraction (integral intensity) of the components (subspectra) used in the fit. A_2/A_3 describes the relative signal intensities of the second and third absorption line of a magnetic hyperfine (6-line) spectrum.

T	δ	ΔE_Q	B_{hf}	Γ_{HWHM}	ν_c	A_2/A_3	$\nu_c B_{hf}^{-1}$	V
4 [a]	0.556(54)	−0.05(9)	49.8(7)	0.44(39)	0.5(4)	4*	0.01(1)	59
	0.413(83)	−0.05(11)	19(1)	0.44*	0.4(2)	4*	0.02(1)	41
90	0.208(3)	−1.38(1)	18.6*	0.157(5)	142(33)	2*	7.6(1.8)	100
4 [b]	0.207(4)	−0.95(10)	18.6(3)	0.12*	0.54(7)	2.5(8)	0.029(4)	59
	0.096(7)	−2.09(10)	18.6*	0.57(17)	16(14)	2*	0.86(75)	41
K	mm s^{-1}	mm s^{-1}	T	mm s^{-1}	mm s^{-1}		mm s^{-1} T^{-1}	%

[a] Recorded after fast cooling to T = 4 K (with a cooling rate $v_{\downarrow}$ > 10 K min^{-1} to access the HS state).

[b] Recorded after a sequence of temperature steps: *i.e.*, (*i*) fast cooling to T = 4 K, (*ii*) warming to T = 90 K and (*iii*) cooling back to T = 4 K to access the LS state.

*) Fixed in the fit.

Note (#1): The ratio A_2/A_3 in Supplementary Table 11 depends on the angle between the internal magnetic field orientation and the γ-ray direction[4] and, therefore, it can be used to describe the average alignment of the electronic magnetic moments of a given sample. In case of the trapped HS state, the fit quality is significantly improved when a ratio of A_2/A_3 = 4 is used, thus indicating a fully polarised sample (*i.e.*, all electronic magnetic moments are aligned in the direction of the applied magnetic field).

Note (#2): The determined volume fractions V are very similar for the trapped HS and the relaxed LS state, implicating that the presence of the two components in these Mössbauer spectra is caused by a similar origin. Since zero-field splitting is in general not possible for S = ½ systems, the two components with different relative fluctuation rates ($\nu_c B_{hf}^{-1}$) in the Mössbauer spectrum of the relaxed LS state are suggesting the presence of two (or more) structural slightly different ^{57}Fe sites that induce this variation (or distribution) of individual relaxation times.

However, in case of the trapped HS state, due to the relatively large line widths even at low temperatures, the identification of all relevant contributions is inherently hampered. Although a fit with two components revealed a good agreement with the experimental data, it cannot safely be ruled out that additional (unresolved) contributions are furthermore present. In particular, the strongly reduced B_{hf} for one of the two identified components in the spectrum of the trapped HS state is indeed implicating that thermally populated $M_S = \pm 3/2$ and $\pm 1/2$ states could be present. Hence, for the trapped HS state, it can be speculated that presumably a combination of zero-field splitting with thermal population of the Kramers states and structural inhomogeneities causes the occurrence of more than one component in the Mössbauer spectrum at T = 4 K.

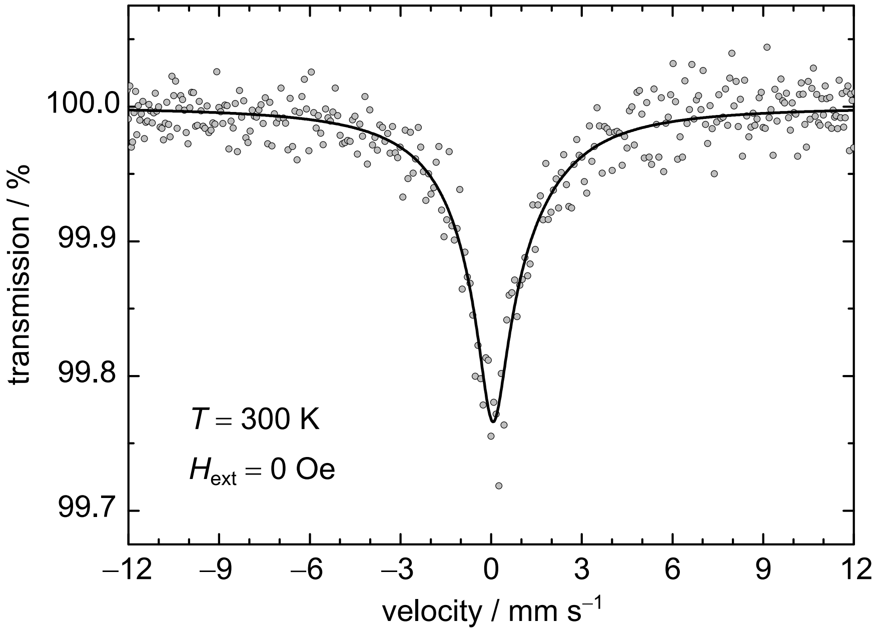

Supplementary Figure 45 | Zero-field Mössbauer spectrum of compound **FeB**, recorded at $T = 300$ K. Symbols: Experimental data. Line: Fit with the Blume-Tjon relaxation model.[17] The parameters of the fit are summarised in Supplementary Table 12.

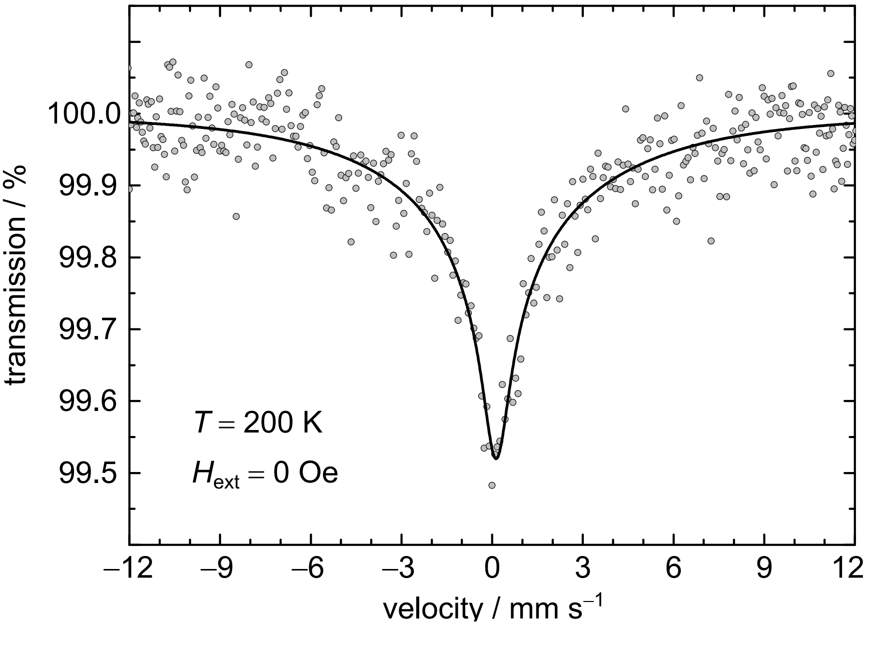

Supplementary Figure 46 | Zero-field Mössbauer spectrum of compound **FeB**, recorded at $T = 200$ K. Symbols: Experimental data. Line: Fit with the Blume-Tjon relaxation model.[17] The parameters of the fit are summarised in Supplementary Table 12.

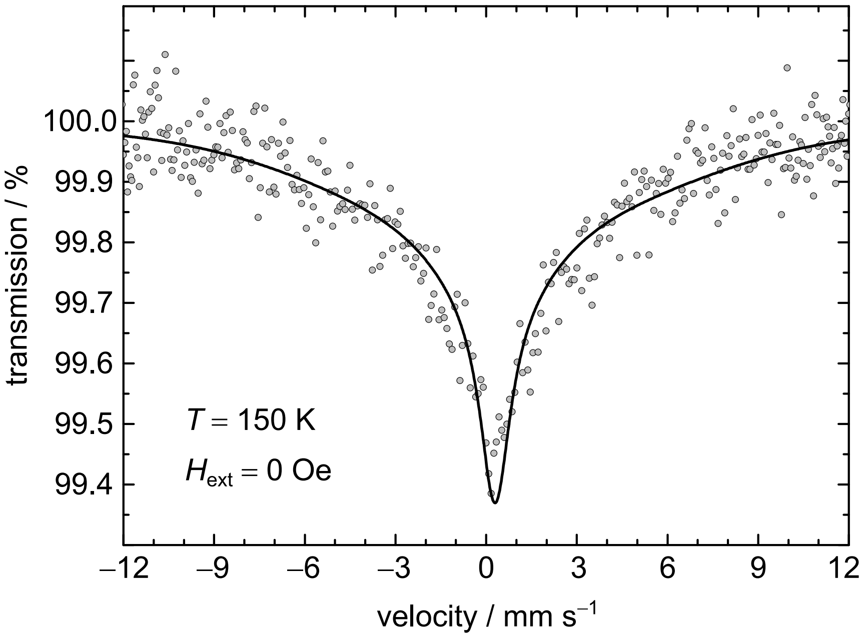

Supplementary Figure 47 | Zero-field Mössbauer spectrum of compound **FeB**, recorded at $T = 150$ K. Symbols: Experimental data. Line: Fit with the Blume-Tjon relaxation model.[17] The parameters of the fit are summarised in Supplementary Table 12.

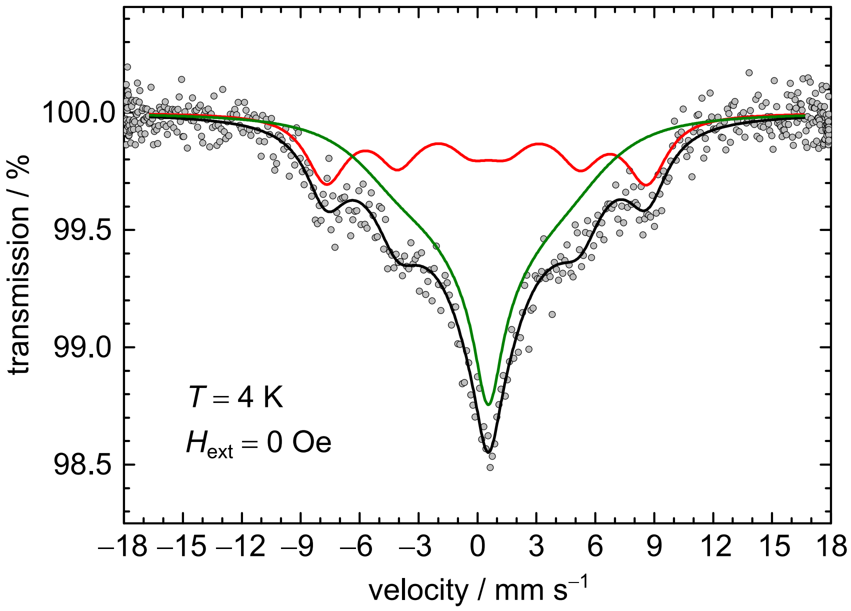

Supplementary Figure 48 | Zero-field Mössbauer spectrum of compound **FeB**, recorded at $T = 4$ K after fast zero-field cooling (with a cooling rate $v\downarrow > 10$ K min^{-1}). Symbols: Experimental data. Lines: Fit with the Blume-Tjon relaxation model.[17] The parameters of the fit are summarised in Supplementary Table 12. In contrast to the measurements at elevated temperatures, two components (red and green) were used in the fit to account for the data. The black line represents the superposition of these two subspectra.

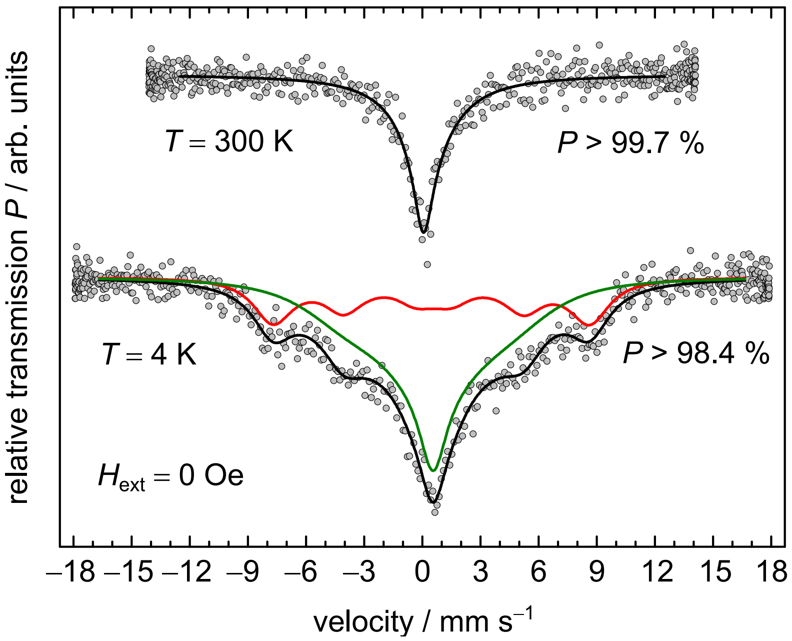

Supplementary Figure 49 | Comparison of the zero-field Mössbauer spectra of compound **FeB**, recorded at T = 4 K (after fast zero-field cooling) and T = 300 K. Here, the same data as shown in Supplementary Figure 45 and Supplementary Figure 48 are displayed. Symbols: Experimental data. Lines: Fit with the Blume-Tjon relaxation model.[17] The parameters of the fit are summarised in Supplementary Table 12.

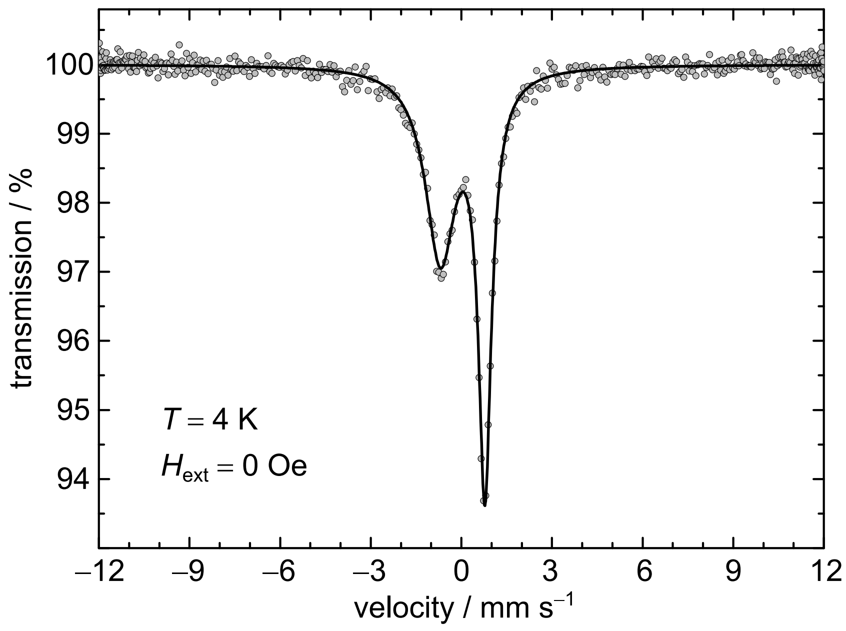

Supplementary Figure 50 | Zero-field Mössbauer spectrum of compound **FeB**, recorded at T = 4 K after a sequence of temperature steps: *i.e.*, (*i*) fast cooling to T = 4 K, (*ii*) warming to T = 90 K and (*iii*) cooling back to T = 4 K. Symbols: Experimental data. Line: Fit with the Blume-Tjon relaxation model.[17] The parameters of the fit are summarised in Supplementary Table 12.

Supplementary Table 12 | Zero-field Mössbauer parameters of compound **FeB**, determined with a fit based on the Blume-Tjon relaxation model.[17] The isomer shift δ is specified relative to metallic iron at room temperature but was not corrected for the second-order Doppler shift. Γ denotes the half (Lorentzian) line widths at half maximum (HWHM), ΔE_Q the quadrupole splitting, ν_c the fluctuation rate of the local magnetic hyperfine field B_{hf} and V the volume fraction (integral intensity) of the components (subspectra) used in the fit.

T	δ	ΔE_Q	B_{hf}	Γ_{HWHM}	ν_c	$\nu_c B_{hf}^{-1}$	V
300	0.31(15)	0.26(40)	51*	0.48(12)	21(7)	0.41(14)	100
200	0.38(7)	0.26*	51*	0.48*	10(2)	0.20(4)	100
150	0.55(6)	0.26*	51*	0.48*	5.9(7)	0.12(1)	100
4 [a]	0.64(14)	–0.12(26)	51(1)	0.74(59)	0.5(7)	0.01(1)	33
	0.56(16)	–0.22(32)	33(5)	0.74*	2.6(7)	0.08(2)	67
90	0.164(3)	–1.38(1)	18.6*	0.142(5)	52(5)	2.80(27)	100
4 [b]	0.163(9)	–1.47(2)	18.6*	0.194(7)	11.2(6)	0.60(3)	100
K	mm s^{-1}	mm s^{-1}	T	mm s^{-1}	mm s^{-1}	mm s^{-1} T^{-1}	%

[a] Recorded after fast cooling to $T = 4$ K (with a cooling rate $v_\downarrow > 10$ K min^{-1}).

[b] Recorded after a sequence of temperature steps: *i.e.*, (*i*) fast cooling to $T = 4$ K, (*ii*) warming to $T = 90$ K and (*iii*) cooling back to $T = 4$ K.

*) Fixed in the fit.

6 FURTHER CHARACTERISATION

6.1 Mass spectra of compound FeB

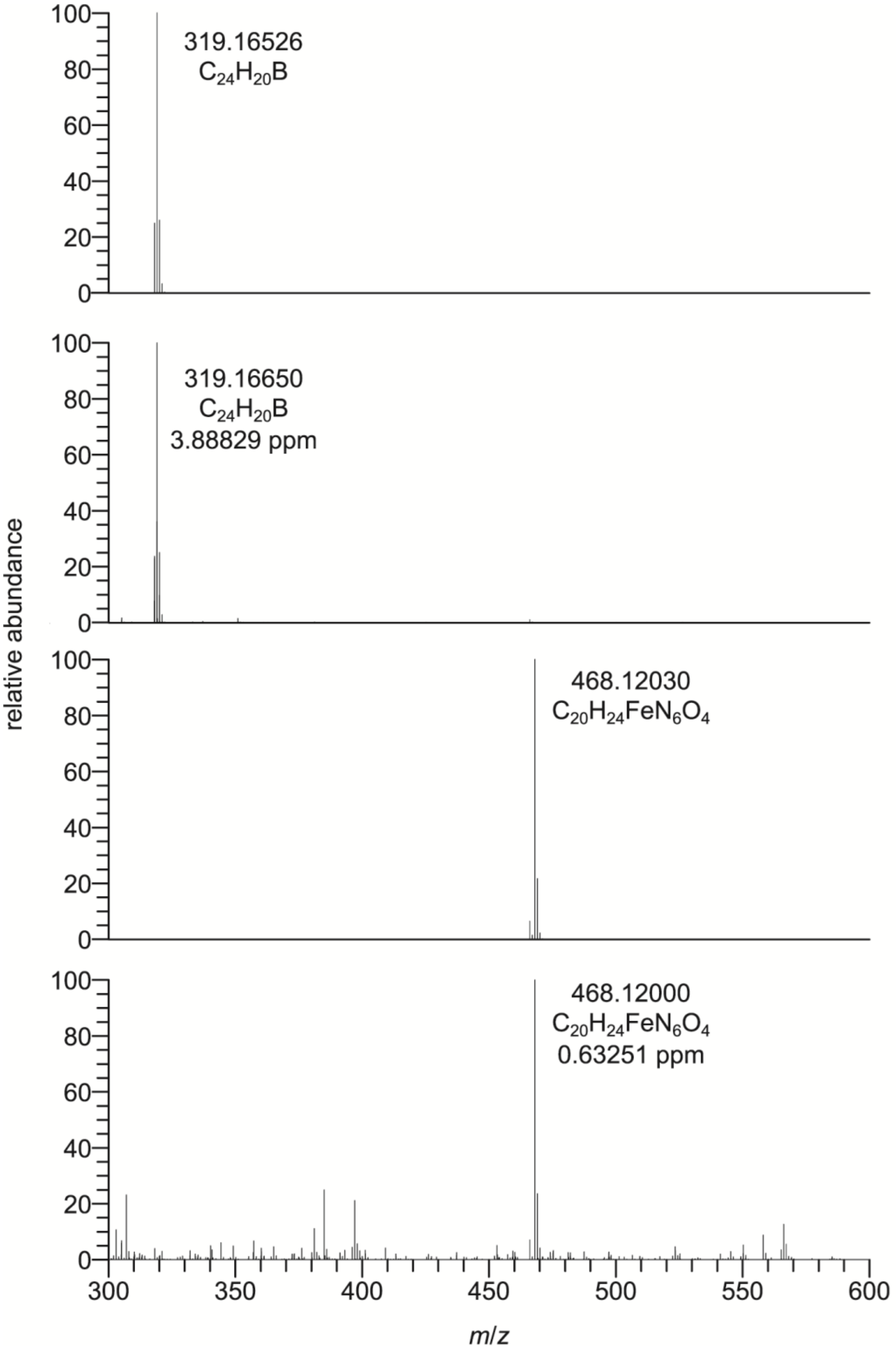

Supplementary Figure 51 | High-resolution mass spectrum of compound **FeB** recorded in the negative (anion, top) and positive (cation, bottom) ESI mode from an acetonitrile/methanol solution.

6.2 IR spectrum of compound FeB

IR spectroscopy was performed at room temperature with a Spectrum One FT-IR spectrometer from PerkinElmer, Inc. with an ATR sampling unit.

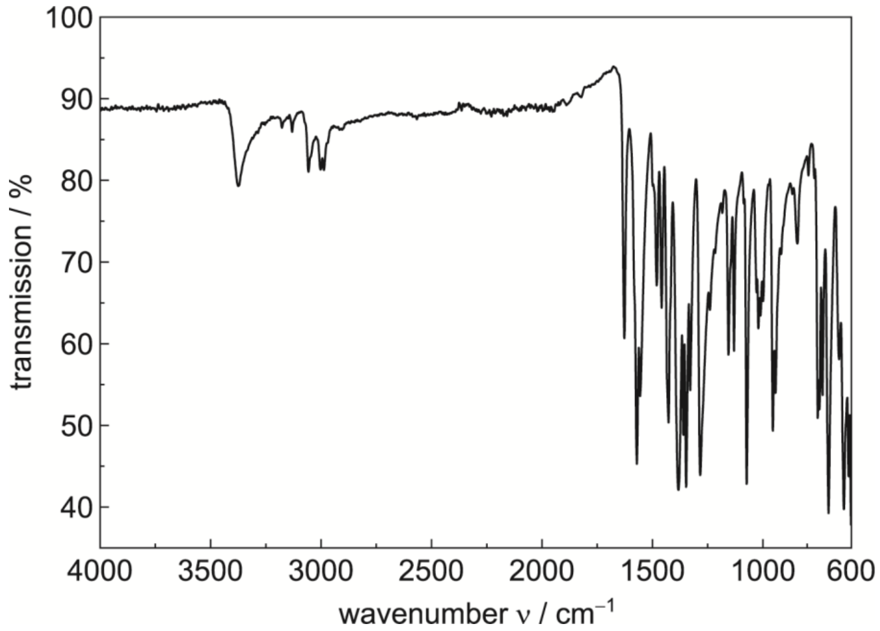

Supplementary Figure 52 | FT-IR (ATR) spectrum of compound **FeB** in the range $\nu = 4000–600\ cm^{-1}$ ($\nu_{max} = 3374$, 3177, 3131, 3057, 3002, 2987, 1627, 1571, 1556, 1481, 1458, 1427, 1383, 1348, 1329, 1284, 1156, 1131, 1074, 1021, 955, 943, 844, 752, 730, 703, 655, 634, 612, 602 cm^{-1}).

6.3 ^{1}H NMR spectrum of ligand HL

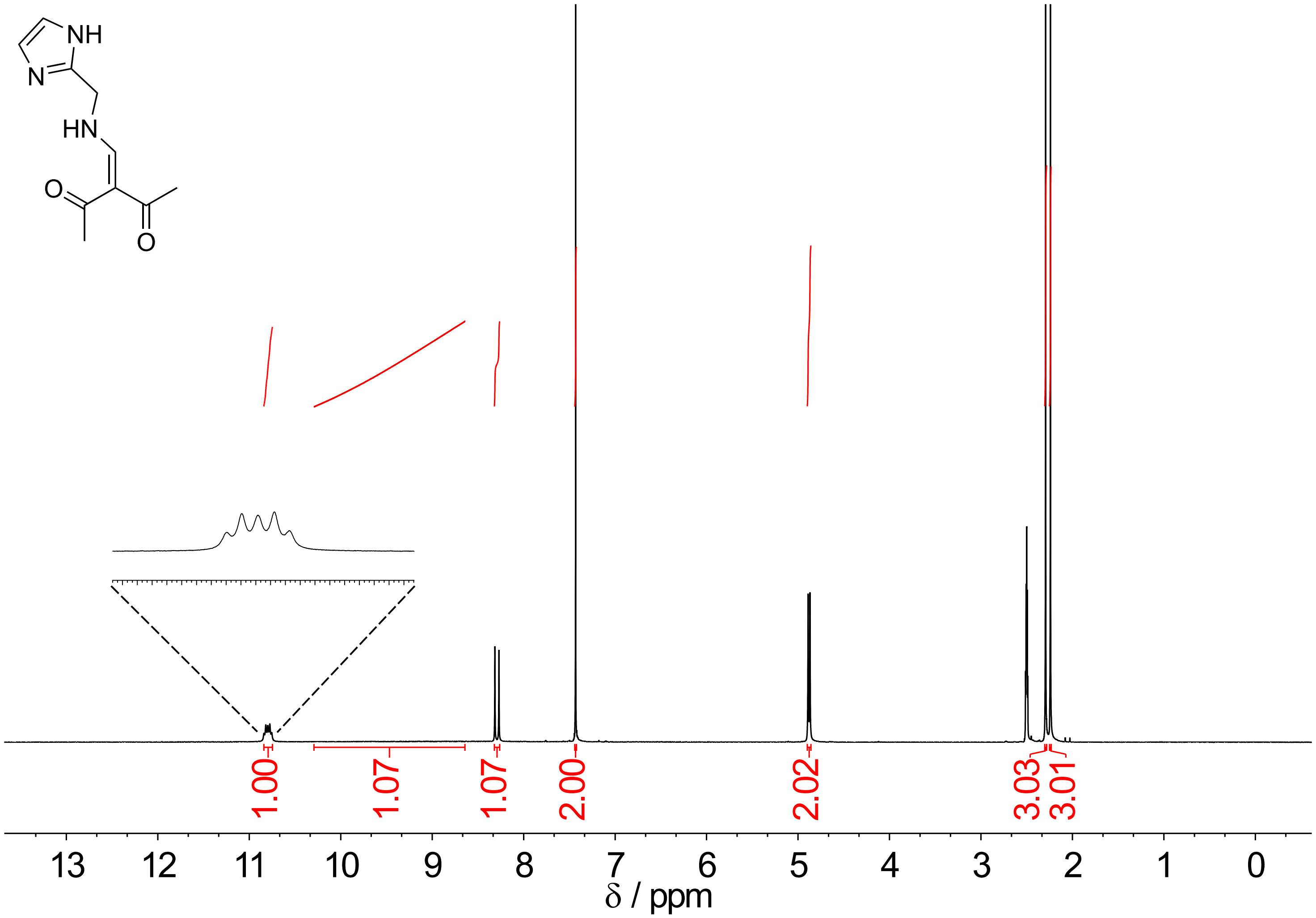

Supplementary Figure 53 | ^{1}H NMR spectrum of HL recorded in DMSO–d_6 at room temperature and $\omega_0 = 300$ MHz.

7 SUPPLEMENTARY REFERENCES

1. Gütlich, P. Physikalische Methoden in der Chemie: Mößbauer-Spektroskopie I. *Chem. unserer Zeit* **4,** 133–144; 10.1002/ciuz.19700040502 (1970).
2. Gütlich, P. Physikalische Methoden in der Chemie: Mößbauer-Spektroskopie II. *Chem. unserer Zeit* **5,** 131–141; 10.1002/ciuz.19710050502 (1971).
3. Greenwood, N. N. & Gibb, T. C. Mössbauer Spectroscopy (Springer, Berlin, 2013).
4. Goldanskii, V. I & Herber, R. H. Chemical applications of Mössbauer spectroscopy (Academic Press, London, 1968).
5. Gütlich, P., Bill, E. & Trautwein, A. Mössbauer spectroscopy and transition metal chemistry. Fundamentals and application (Springer, Berlin, 2011).
6. Weber, B. *Coordination Chemistry* (Springer, Berlin, 2023).
7. Bastiaansen, L. A. M. & Godefroi, E. F. 2-Aminomethylimidazole and imidazole-2-carboxaldehyde: two facile syntheses. *J. Org. Chem.* **43,** 1603–1604; 10.1021/jo00402a032 (1978).
8. Lázaro Martínez, J. M., Romasanta, P. N., Chattah, A. K. & Buldain, G. Y. NMR characterization of hydrate and aldehyde forms of imidazole-2-carboxaldehyde and derivatives. *J. Org. Chem.* **75,** 3208–3213; 10.1021/jo902588s (2010).
9. Ketkaew, R. *et al.* OctaDist: a tool for calculating distortion parameters in spin crossover and coordination complexes. *Dalton Trans.* **50,** 1086–1096; 10.1039/d0dt03988h (2021).
10. Spackman, M. A. & Jayatilaka, D. Hirshfeld surface analysis. *CrystEngComm* **11,** 19–32; 10.1039/B818330A (2009).
11. Spackman, P. R. *et al.* CrystalExplorer: a program for Hirshfeld surface analysis, visualization and quantitative analysis of molecular crystals. *J. Appl. Crystallogr.* **54,** 1006–1011; 10.1107/S1600576721002910 (2021).
12. Halcrow, M. A. Structure:function relationships in molecular spin-crossover complexes. *Chem. Soc. Rev.* **40,** 4119–4142; 10.1039/c1cs15046d (2011).
13. Halcrow, M. A. Iron(II) complexes of 2,6-di(pyrazol-1-yl)pyridines—A versatile system for spin-crossover research. *Coord. Chem. Rev.* **253,** 2493–2514; 10.1016/j.ccr.2009.07.009 (2009).
14. Macrae, C. F. *et al.* Mercury 4.0: from visualization to analysis, design and prediction. *J. Appl. Cryst.* **53,** 226–235; 10.1107/S1600576719014092 (2020).

15. Delgado, T. *et al.* Very Long-Lived Photogenerated High-Spin Phase of a Multistable Spin-Crossover Molecular Material. *J. Am. Chem. Soc.* **140,** 12870–12876; 10.1021/jacs.8b06042 (2018).

16. Hauser, A. Cooperative effects on the HS→LS relaxation in the $[Fe(ptz)_6](BF_4)_2$ spin-crossover system. *Chem. Phys. Lett.* **192,** 65–70; 10.1016/0009-2614(92)85429-E (1992).

17. Blume, M. & Tjon, J. A. Mössbauer Spectra in a Fluctuating Environment. *Phys. Rev.* **165,** 446–456; 10.1103/PhysRev.165.446 (1968).
